# A life that’s worth living – measuring health-related quality of life among people treated for tuberculosis in Viet Nam: a longitudinal EQ-5D-5L survey

**DOI:** 10.1186/s12955-025-02369-9

**Published:** 2025-04-23

**Authors:** Luan Nguyen Quang Vo, Rachel Forse, Andrew James Codlin, Huy Ba Huynh, Anja Maria Christine Wiemers, Jacob Creswell, Tushar Garg, Thi Minh Ha Dang, Lan Huu Nguyen, Hoa Binh Nguyen, Luong Van Dinh, Nhung Viet Nguyen, Tom Wingfield, Kristi Sidney Annerstedt, Jad Shedrawy, Knut Lönnroth

**Affiliations:** 1 Friends for International TB Relief, Ha Noi, Viet Nam; 2 Department of Global Public Health, Karolinska Institutet, Stockholm, Sweden; 3 Stop TB Partnership, Geneva, Switzerland; 4 Pham Ngoc Thach Hospital, Ho Chi Minh City, Viet Nam; 5 National Lung Hospital, Ha Noi, Viet Nam; 6 VNU University of Medicine and Pharmacy, Ha Noi, Viet Nam; 7 Centre for Tuberculosis Research, Departments of Clinical Sciences and International Public Health, Liverpool School of Tropical Medicine, Liverpool, UK; 8 Tropical and Infectious Diseases Unit, Liverpool University Hospital NHS Foundation Trust, Liverpool, UK

**Keywords:** Tuberculosis, Patient-reported outcome measures, Health-related quality of life, EQ-5D-5L, Viet Nam, Longitudinal, PPM, Active case finding

## Abstract

**Background:**

In many settings, Tuberculosis (TB) represents a catastrophic life event that substantially impairs a person’s Health-Related Quality of Life (HRQoL). We aimed to measure HRQoL among people with TB in Viet Nam at initiation and throughout treatment.

**Methods:**

This study took place in four provinces from Oct-2020 to Sep-2022. Persons initiated on TB treatment were consecutively recruited across three pathways to access care: passive case finding (NTP); active case finding (ACF); and private sector engagement (PPM). We conducted the EuroQol–5-Dimension–5-Level (EQ-5D-5L) survey during the intensive, continuation, and post-treatment phase. We described participant characteristics, assessed the survey’s psychometric properties, and calculated utility indexes using a Vietnamese value set. We reported these alongside visual analog scale (EQ-VAS) scores and EQ-5D-5L dimensions by treatment stage, care pathway and other participant characteristics. Mixed-effect Tobit models were fitted to identify relevant associations with HRQoL, which we compared to general population benchmarks.

**Results:**

We recruited 585 participants (23.6% female) with a median age of 51 years. EQ-5D-5L dimensions at baseline showed that 53.8% experienced *pain/discomfort* and 35.0% felt *anxiety/depression*, while 33.8%, 30.4%, and 9.6% reported problems with carrying out *usual activities*, *mobility*, and *self-care*, respectively. The mean utility index was 0.83 (95% confidence interval: [0.82, 0.85]) and mean EQ-VAS was 67.1 (95%CI: [65.6, 68.6]). Post-treatment, HRQoL improved significantly on all dimensions and composite measures. While utility indexes were at parity with general population benchmarks (0.90; 95%CI: [0.89, 0.92] vs. 0.91), self-reported EQ-VAS scores remained significantly lower (79.4; 95%CI: [78.1, 80.6] vs. 87.4). HRQoL was higher at baseline in the ACF versus the NTP cohorts on utility index (0.87 vs. 0.82; *p* = 0.003) and EQ-VAS score (70.4 vs. 65.5; *p* = 0.015). The EQ-5D-5L tool demonstrated moderate to high validity on Cronbach’s alpha (0.75 ≤ α ≤ 0.84) and Spearman’s rho (0.4679 ≤ *ρ*_0_ ≤ 0.5651) across treatment stages and various known groups.

**Conclusion:**

TB significantly impairs HRQoL among affected Vietnamese people. While treatment partially remedies these impairments, they may persist post-TB. Hence, physical, psychological and social rehabilitation during and after therapy should receive more attention. We found evidence that ACF may mitigate TB-related declines in HRQoL, but tailored studies are needed to substantiate these findings.

**Supplementary Information:**

The online version contains supplementary material available at 10.1186/s12955-025-02369-9.

## Background

One billion people have died from tuberculosis (TB) in the past 200 years [1]. With timely and appropriate treatment, 86% of TB-affected persons are cured globally [2]. Each year, about nine million people live with or beat the disease, including 171,000 individuals with TB in Viet Nam who are successfully treated [3]. However, surviving TB does not mean life resumes seamlessly. TB-affected persons frequently experience disability despite successful treatment [4]. Beyond clinical issues, an episode of TB also carries grave economic, psychological and social consequences [5–10]. 

To assess this multifactorial impact, researchers have increasingly relied on patient-reported outcome measures such as health-related quality of life (HRQoL) [11–13]. The EuroQoL–5-Dimension–5-Level (EQ-5D-5L) tool has demonstrated reliable psychometric properties, and thus been used in many settings [14]. In Viet Nam the tool was used to measure HRQoL in the general public and sub-populations such as the elderly and ethnic minorities [15–17]. Disease-specific studies included cancer [18, 19], cardiovascular disease [20], diabetes [21], chronic respiratory diseases [22], mental illness [23], HIV/AIDS [24, 25], and COVID-19 [26, 27]. One notable exception is TB, for which there are few local studies on HRQoL and none conducted longitudinally. This knowledge gap is in stark contrast with the annual 172,000 persons falling ill with TB and 13,600 TB-related mortalities, and the high costs borne by TB-affected households [28, 29]. 

In other high-burden settings, EQ-5D-5L surveys have found that TB significantly impairshealth-related quality of life, often in conjunction with the presentation of characteristic symptoms such as cough, fever, night sweats and unexplained weight loss [13]. While TB treatment can improve HRQoL [30, 31], it generally does not fully recover even when successfully treated, commonly due to post-TB sequelae [32–35]. Conversely, there are also known factors that may be associated with higher HRQoL in TB-affected persons such as male sex, younger age, and higher levels of education and income [13]. 

The pathway by which TB care is accessed and the environment in which it is provided may affect HRQoL. Active case finding (ACF) and private sector engagement, denoted by the World Health Organization (WHO) as public-privatemix (PPM), are two strategies by which persons with TB can receive treatment earlier and under preferential conditions compared to passive case finding (PCF) [36–40]. ACF has been attributed with early detection, which may be linked to better health outcomes [41–43]. The same applies to private sector care, which also offers better confidentiality and convenience [44–46]. These benefits can translate to reduced stigma [47], which in turn has been linked to higherhealth-related quality of life [48]. Yet, few studies have measured the impact of different care pathways on HRQoL. One study from Nepal did not detect a difference between ACF and PCF [49]. A study in Indonesia included different PPM pathways, but did not compare HRQoL between them [48]. Lastly, a Ugandan study found higher HRQoL in people with TB receiving care in private hospitals compared to PCF, but did not use the EQ-5D-5L [50]. 

This study’s primary objective was to measure HRQoL among people with TB in Viet Nam at initiation and throughout treatment. As secondary objective, our study tested the hypothesis that persons with TB detected through ACF and PPM have a higher HRQoL at baseline than under routine care offered by the NTP. Lastly, our tertiary objective was to assess the psychometric properties of the EQ-5D-5L tool in this setting and target population.

## Methods

### Study design

This cohort study measured EQ-5D-5L dimensions, utility indexes and EQ-VAS in persons with TB in Viet Nam at three treatment milestones and accessing TB services through three distinct pathways.

### Setting

**Fig. 1:**
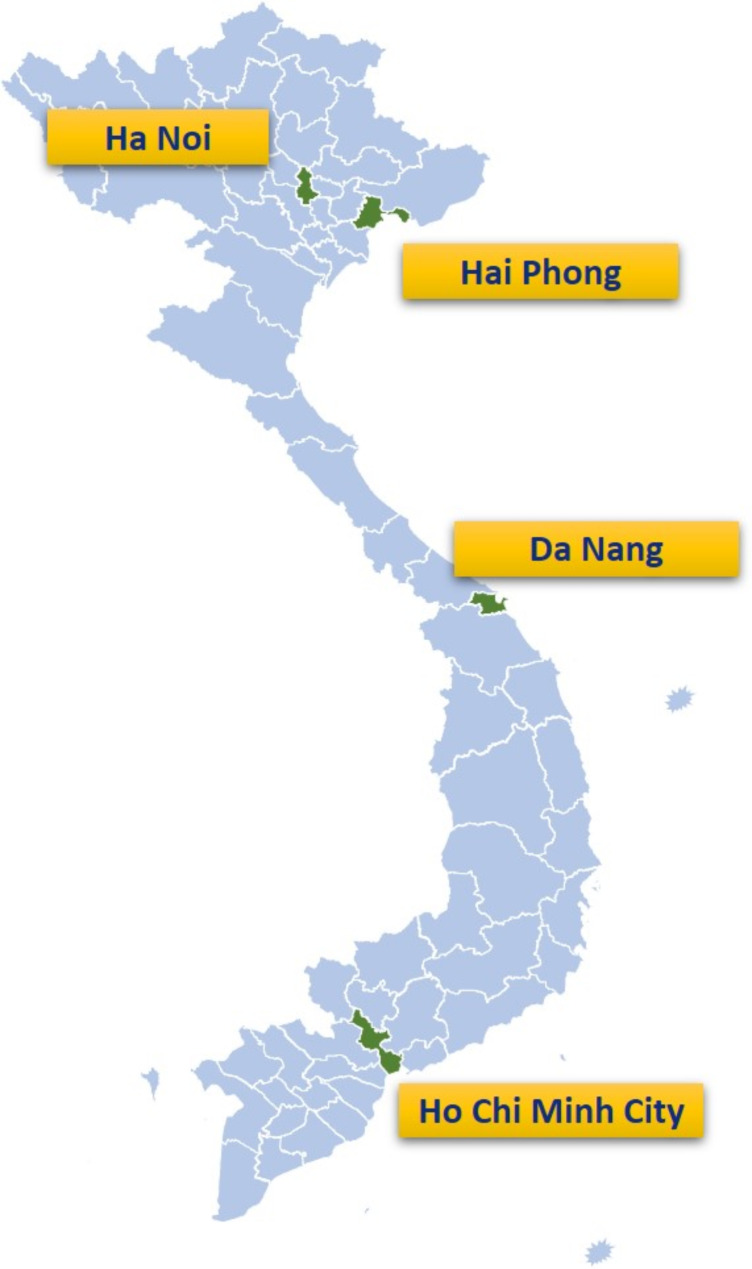
Map of study provinces

The study was conducted in 28 districts in Ha Noi, Hai Phong, Da Nang and Ho Chi Minh City (HCMC), Viet Nam (Fig. 1). These provinces had a population of 20.2 million [51]. In 2022, the NTP notified 26,822 persons with drug-susceptible TB (DS-TB). The overall treatment success rate was 87.9% [52, 53]. The male-to-female ratio was 4.0 and the rate of catastrophic cost, defined as cost incurrence equivalent to ≥ 20% of annual household income due to the episode of TB, was 21.9–57.5% [54–58]. 

### Study population and eligibility

The sampling frame consisted of people initiating DS-TB treatment with the NTP or a private provider. Participants in the ACF cohort were identified from ACF event records and recruited after treatment initiation with the NTP. Private sector participants were screened and referred by the private provider. We included persons aged ≥ 18 years with pulmonary DS-TB living in the study provinces and providing informed consent. Persons with permanent residency outside of the study provinces were excluded.

### Data sources & collection

Our data were sourced from four separate studies that consecutively recruited and followed participants from October 2020 to September 2022. While sampling differed to meet the specific aims of those studies, all employed a longitudinal design, and followed the same data collection methodology using the EQ-5D-5L tool. Interviews included a localized version of the WHO patient cost survey (PCS) detailed elsewhere [55, 56, 59]. The sample size was guided by available funds to meet sponsor requirements, rather than to measure a specific HRQoL-related endpoint.

The EQ-5D-5L instrument was chosen based on its prior utilization and validation in Viet Nam among the general population and key sub-groups [14, 25, 60]. Briefly, the tool assesses HRQoL along five dimensions including *mobility*, *self-care*, and *carrying out activities as usual*, e.g., work/study, housework, family, and leisure activities [61], as well as experiencing *pain/discomfort* and *anxiety/depression* using five levels of health states. The instrument includes a visual analog scale (EQ-VAS) to self-rate overall wellbeing from 0 to 100.

**Fig. 2:**
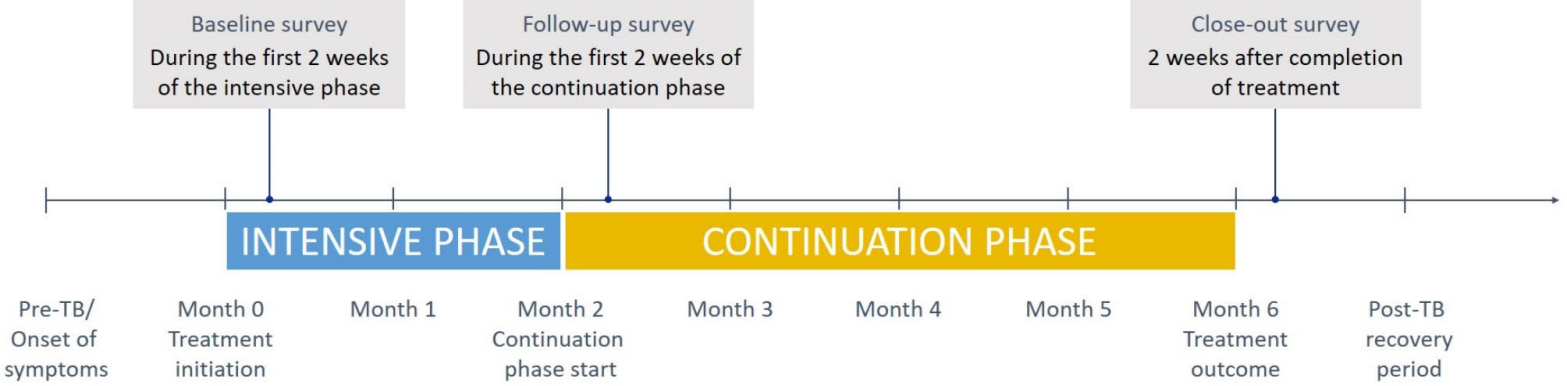
Timeline of longitudinal surveys

Participants were surveyed thrice (Fig. 2) within three weeks of key milestones: (1) TB treatment initiation (intensive phase or baseline); (2) mid-treatment upon entering the continuation phase (CP); and (3) end of treatment (EOT). Interviews were conducted in-person by trained staff at TB care facilities or participant homes, or by phone during COVID-19-related restrictions [62]. Data were collected on paper and via audio recording. Paper surveys were digitized and 5% were randomly selected for verification against the audio recordings and paper forms.

Clinical and socioeconomic covariates were abstracted from NTP registers and PCS surveys, respectively. Financial data were collected in Viet Nam Dong (VND) and converted to United States Dollar (USD) using average exchange rates for the study period (VND 1 = USD 0.000043; XE.com). Clinical predictors were bifurcated and included diagnosis (bacteriologically-confirmed/clinically diagnosed), disease site (pulmonary/extra-pulmonary), treatment category (new/retreatment) and outcome (treatment success/unfavorable outcome). Treatment success entailed persons who were cured or completed treatment, while unfavorable outcomes included failure, loss to follow-up, transfer out and death as per standard WHO definitions. Socioeconomic data encompassed level and duration of education, role in the household (head, primary earner or otherwise), monthly pre-TB earnings, post-TB income loss, job loss and financial coping mechanisms accessed.

### Statistical analyses

We calculated descriptive statistics of demographic, clinical, and socioeconomic characteristics as well as health access and social impact of TB.

We tabulated EQ-5D-5L responses for each dimension and the top 10 health states, i.e., the concatenated response patterns of the five EQ-5D-5L dimensions with the *11111* pattern representing perfect health, by treatment stage. Each dimension was dichotomized into participants with and without any impairment. Differences were assessed using the Mann-Whitney tests for ordinal matched pairs. We calculated EQ-5D-5L utility indexes using a Viet Nam-specific value set [63]. The composite measures of utility index and EQ-VAS score were tabulated by treatment stage and participant covariates. We compared the EQ-5D-5L dimensions, utility indexes and EQ-VAS scores to Viet Nam’s general population and the sub-group aged 45–54 years based on our sample’s median age [15]. We identified differences using 1-sample z-tests of proportions and Wilcoxon signed-rank tests.

To assess longitudinal changes in utility indexes and EQ-VAS scores, we used two-level repeated measures (RM-)ANOVA with an interaction term between treatment stage and the exposure of interest, or Friedman’s test if RM-ANOVA residuals were non-normally distributed. When RM-ANOVA was used, we assessed sphericity using the Mauchly test and addressed violations using Greenhouse-Geisser correction or Huynh-Feldt adjustments when corrective factors were ≥ 0.6 [64]. Differences within participant covariates in each treatment stage were identified using Mann-Whitney and Kruskal-Wallis tests.

For comparison across care pathways, we provided reference values of a Minimum Clinically Important Difference (MCID), which has been cited to link statistical testing with real-world clinical significance [65]. As no TB-specific MCID values for Viet Nam were available, we used reference values for persons with one health issue in the general population of 0.07 and 9.8 for utility indexes and EQ-VAS scores, respectively [15]. We fitted mixed-effect, saturated, Tobit models with robust standard errors and study province as the random effect to obtain adjusted coefficients of utility indexes (β_UI_) and EQ-VAS scores (β_VAS_) at CP and EOT (denoted by subscripts) in a complete-case analysis.

To evaluate the EQ-5D-5L tool’s psychometric properties among persons with TB in Viet Nam, we estimated internal consistency using Cronbach’s alpha, grading *α* ≥ 0.7 as reliable. We assessed convergent validity between the utility index and EQ-VAS scores using Spearman’s coefficient for each treatment stage, classified as weak (*ρ*_0_ < 0.3), moderate (0.3 ≤ *ρ*_0_ < 0.5); and strong (≥ 0.5) [15]. We measured discriminative validity, i.e., degree to which known groups with differing HRQoL can be identified, using Mann-Whitney and Kruskal-Willis tests with a pairwise post-hoc comparison for the latter using Dunn’s test with the Benjamini-Hochberg adjustment [66]. 

Analyses were conducted using Stata v17 (Statacorp; College Station, TX). Hypothesis tests were two-tailed and *p* ≤ 0.05 was considered significant. Results were reported according to the STROBE statement for cohort studies. We included participant characteristics, EQ-5D-5L dimensions, utility indexes and EQ-VAS disaggregated by care pathway in the supplementary information (Tables S1, S2 and S4a, b and c).

## Results

### Participant characteristics

Of 1,535 persons evaluated for enrollment, 57.8% (887/1,535) were ineligible and 2.6% (40/1,535) declined to participate. Of 608 enrolled, 3.8% (23/608) withdrew or were lost to follow-up. The final study sample included 585 participants who completed all three surveys and were analyzed (Supplementary information, Figure S1). The median age was 51 years (Interquartile range [IQR]: 36–60) and 23.6% (138/585) were female (Table 1). Overall, 73.3% (429/585) belonged to the NTP cohort, 18.1% (106/585) the ACF cohort, and 8.6% (50/585) the PPM cohort. Persons with bacteriologically-confirmed TB accounted for 93.5% (545/583), 98.6% (575/583) had pulmonary TB, 82.2% (479/583) were treatment-naïve and 95.5% (507/531) completed treatment successfully.

**Table 1:** Participant characteristics

	*N*	%
**Total**	585	100.0
**DEMOGRAPHICS**		
Sex		
Female	138	23.6
Male	447	76.4
Age		
<35 years	129	22.1
35–44 years	98	16.8
45–54 years	113	19.3
55–64 years	155	26.5
65 + years	90	15.4
* Participant age (median*,* IQR)*	*51*	*(36–60)*
Province		
Ha Noi	55	9.4
Hai Phong	76	13.0
Ho Chi Minh City	434	74.2
Da Nang	20	3.4
**CLINICAL CHARACTERISTICS**		
Diagnosis (*N* = 583)		
Clinically diagnosed	38	6.5
Bacteriologically-confirmed	545	93.5
TB site (*N* = 583)		
Pulmonary TB	575	98.6
Extrapulmonary TB	8	1.4
Treatment category (*N* = 583)		
New	479	82.2
Retreatment	104	17.8
Treatment outcome (*N* = 531)		
Treatment success	507	95.5
Unfavorable outcome*	24	4.5
**HEALTH ACCESS**		
Care pathway		
National TB Program	429	73.3
Active Case Finding	106	18.1
Public-Private-Mix	50	8.6
Diagnostic delay (*N* = 515)		
1–3 weeks	90	17.5
4–5 weeks	86	16.7
6–10 weeks	120	23.3
11–21 weeks	113	21.9
22 + weeks	106	20.6
* Diagnostic delay (median*,* IQR)*	*9*	*(4–18)*
Health-seeking		
1–2 attempts	78	13.3
3–4 attempts	149	25.5
5–6 attempts	115	19.7
7–10 attempts	120	20.5
11 + attempts	123	21.0
* Health-seeking attempts (median*,* IQR)*	*6*	*(3–10)*
Social Health Insurance		
No	133	22.7
Yes	452	77.3
**SOCIOECONOMIC CHARACTERISTICS¥**		
Education level		
Not literate	25	4.3
Primary school	213	36.4
Secondary school	156	26.7
High school	111	19.0
University/Post-graduate	80	13.7
Education length		
0–4 years	108	18.5
5–6 years	98	16.8
7–8 years	93	15.9
9–11 years	122	20.9
12 + years	164	28.0
* Years of education (median*,* IQR)*	*8*	*(5–12)*
Head of household		
No	252	43.1
Yes	333	56.9
Household size		
1 person	44	7.5
2 persons	91	15.6
3 persons	114	19.5
4 persons	142	24.3
5 + persons	194	33.2
* Household size (median*,* IQR)*	*4*	*(3–5)*
Primary income earner of household		
No	321	54.9
Yes	264	45.1
Employment		
Unemployed	167	28.6
Formally employed	77	13.2
Informally employed	267	45.6
Don’t know/No answer	74	12.7
Pre-TB monthly income		
USD 0–24	117	20.0
USD 25–169	117	20.0
USD 170–259	125	21.4
USD 260–389	115	19.7
USD 390+	111	19.0
* Pre-TB monthly income (median*,* IQR)*	*213*	*(64–341)*
**SOCIOECONOMIC IMPACT OF TB**		
Monthly income decline		
No decline	241	41.2
USD 1-100	94	16.1
USD 101–250	93	15.9
USD 251–400	93	15.9
USD 401+	64	10.9
* Loss in monthly income (median*,* IQR)*	*64*	*(0-255)*
Job loss		
No	472	80.7
Yes	113	19.3
Borrow or receive cash		
No	425	72.7
Yes	160	27.4
Sell assets		
No	555	94.9
Yes	30	5.1

Notes: TB = Tuberculosis; IQR = Interquartile Range; * Unfavorable outcomes include treatment failure, loss to follow-up, transfer out and death; ¥ At baseline

### HRQoL at initiation, mid-treatment and after TB treatment completion

At treatment initiation, the dimension with the highest rate of impairment was *pain/discomfort* (53.8%), followed by *anxiety/depression* (35.0%), *usual activities* (33.8%) and *mobility* (30.4%). Only 9.6% reported problems in *self-care* (Table 2). Mid-treatment, 44.6% and 29.2% of participants reported any *pain/discomfort* and *anxiety/depression*, respectively, while the respective rates of impairment on *usual activities*, *mobility* and *self-care* were 23.2%, 24.5% and 9.9%. After treatment, there were significant improvements on all dimensions of + 12.1 to + 24.4% points (pp) except *self-care* (2.1pp; *p* = 0.361). Across all dimensions, this change was driven by a reduction in people with slight complaints to no problems. The perfect health response (Table 3) at baseline was reported by 28.9% of participants, including 26.3% in the NTP cohort, 38.7% in the ACF cohort and 30.0% in the PPM cohort. Post-treatment, 57.6% of respondents reported perfect health, which was highest in the PPM cohort (68.0%) and lowest in the ACF cohort (53.8%).

**Table 2:** EQ-5D-5L responses by stage of treatment

	Intensive phase		Continuation phase		End of treatment			
	N	%	N	%	N	%	Δ^¥^	*p*-value^¶^
**MOBILITY**								
No problems	407	69.6	442	75.6	494	84.4	+ 14.8	< 0.001
Any problems	178	30.4	143	24.5	91	15.6	-14.8	
Slight problems	132	22.6	103	17.6	54	9.2	-13.4	
Moderate problems	15	2.6	19	3.3	16	2.7	+ 0.1	
Severe problems	28	4.8	19	3.3	18	3.1	-1.7	
Unable to walk about	3	0.5	2	0.3	3	0.5	0.0	
**SELF-CARE**								
No problems	529	90.4	527	90.1	541	92.5	+ 2.1	0.361
Any problems	56	9.6	58	9.9	44	7.5	-2.1	
Slight problems	41	7.0	43	7.4	27	4.6	-2.4	
Moderate problems	5	0.9	7	1.2	6	1.0	+ 0.1	
Severe problems	7	1.2	3	0.5	7	1.2	0.0	
Unable to wash or dress myself	3	0.5	5	0.9	4	0.7	+ 0.2	
**USUAL ACTIVITIES**								
No problems	387	66.2	449	76.8	510	87.2	+ 21.0	< 0.001
Any problems	198	33.8	136	23.2	75	12.8	-21.0	
Slight problems	128	21.9	98	16.8	40	6.8	-15.1	
Moderate problems	22	3.8	13	2.2	12	2.1	-1.7	
Severe problems	26	4.4	14	2.4	15	2.6	-1.8	
Unable to do	22	3.8	11	1.9	8	1.4	-2.4	
**PAIN/DISCOMFORT**								
No pain	270	46.2	324	55.4	413	70.6	+ 24.4	< 0.001
Any pain	315	53.8	261	44.6	172	29.4	-24.4	
Slight pain	227	38.8	175	29.9	117	20.0	-18.8	
Moderate pain	43	7.4	45	7.7	21	3.6	-3.8	
Severe pain	42	7.2	36	6.2	32	5.5	-1.7	
Extreme pain	3	0.5	5	0.9	2	0.3	-0.2	
**ANXIETY/DEPRESSION**								
Not anxious or depressed	380	65.0	414	70.8	451	77.1	+ 12.1	< 0.001
Any anxiety or depression	205	35.0	171	29.2	134	22.9	-12.1	
Slightly	127	21.7	106	18.1	80	13.7	-8.0	
Moderately	34	5.8	33	5.6	26	4.4	-1.4	
Severely	42	7.2	27	4.6	22	3.8	-3.4	
Extremely	2	0.3	5	0.9	6	1.0	+ 0.7	

Notes: ¥ Frequency difference between the Intensive Phase and End of Treatment; ¶ Wilcoxon signed-rank test

**Table 3:** Top 10 response patterns of health states by care pathway and stage of treatment

Intensive phase				Continuation phase				End of treatment			
Pattern^¥^	N	%	Σ%	Pattern^¥^	N	%	Σ%	Pattern^¥^	N	%	Σ%
***Total*** (*N* = *585*)											
11111	169	28.9	28.9	11111	224	38.3	38.3	11111	337	57.6	57.6
11121	79	13.5	42.4	11121	56	9.6	47.9	11121	46	7.9	65.5
11112	29	5.0	47.4	11112	30	5.1	53.0	11112	34	5.8	71.3
11122	24	4.1	51.5	11122	24	4.1	57.1	11122	20	3.4	74.7
21121	22	3.8	55.2	11211	16	2.7	59.8	11113	8	1.4	76.1
11211	20	3.4	58.6	21121	16	2.7	62.6	21111	8	1.4	77.4
11221	14	2.4	61.0	11221	12	2.1	64.6	21121	8	1.4	78.8
21111	11	1.9	62.9	11131	11	1.9	66.5	11141	7	1.2	80.0
21221	11	1.9	64.8	21111	9	1.5	68.0	11123	4	0.7	80.7
21222	11	1.9	66.7	11113	7	1.2	69.2	21222	4	0.7	81.4
***NTP ***(*N* = *429*)											
11111	113	26.3	26.3	11111	160	37.3	37.3	11111	246	57.3	57.3
11121	59	13.8	40.1	11121	42	9.8	47.1	11121	33	7.7	65.0
11112	20	4.7	44.8	11112	21	4.9	52.0	11112	24	5.6	70.6
21121	19	4.4	49.2	11122	19	4.4	56.4	11122	17	4.0	74.6
11122	18	4.2	53.4	21121	13	3.0	59.5	21121	8	1.9	76.4
11211	12	2.8	56.2	11211	10	2.3	61.8	11113	7	1.6	78.1
21222	10	2.3	58.5	11221	10	2.3	64.1	21111	7	1.6	79.7
21221	9	2.1	60.6	11131	9	2.1	66.2	11123	4	0.9	80.6
11221	8	1.9	62.5	21111	7	1.6	67.8	11141	4	0.9	81.6
21111	8	1.9	64.3	11141	5	1.2	69.0	11114	3	0.7	82.3
***ACF*** (*N* = *106*)											
11111	41	38.7	38.7	11111	44	41.5	41.5	11111	57	53.8	53.8
11121	12	11.3	50.0	11112	3	2.8	44.3	11121	10	9.4	63.2
11211	6	5.7	55.7	11113	2	1.9	46.2	11112	9	8.5	71.7
11112	5	4.7	60.4	11114	1	0.9	47.2	11122	2	1.9	73.6
11122	4	3.8	64.2	11121	9	8.5	55.7	11141	2	1.9	75.5
11221	4	3.8	67.9	11122	4	3.8	59.4	11124	1	0.9	76.4
21121	3	2.8	70.8	11124	1	0.9	60.4	11211	1	0.9	77.4
12121	2	1.9	72.6	11131	2	1.9	62.3	11212	1	0.9	78.3
21111	2	1.9	74.5	11132	1	0.9	63.2	12111	1	0.9	79.2
21232	2	1.9	76.4	11141	1	0.9	64.1	12112	1	0.9	80.2
***PPM ***(*N* = *50*)											
11111	15	30.0	30.0	11111	20	40.0	40.0	11111	34	68.0	68.0
11121	8	16.0	46.0	11112	6	12.0	52.0	11121	3	6.0	74.0
11112	4	8.0	54.0	11121	5	10.0	62.0	11112	1	2.0	76.0
11122	2	4.0	58.0	11211	3	6.0	68.0	11113	1	2.0	78.0
11211	2	4.0	62.0	11221	2	4.0	72.0	11122	1	2.0	80.0
11221	2	4.0	66.0	12111	2	4.0	76.0	11141	1	2.0	82.0
21221	2	4.0	70.0	11113	1	2.0	78.0	11243	1	2.0	84.0
11113	1	2.0	72.0	11122	1	2.0	80.0	12123	1	2.0	86.0
11123	1	2.0	74.0	11123	1	2.0	82.0	12211	1	2.0	88.0
11213	1	2.0	76.0	11222	1	2.0	84.0	21111	1	2.0	90.0

Notes: Indicates concatenated responses for the level of health state in ascending order of impairment for the five EQ-5D-5L dimensions in the order of mobility, self-care, usual activities, pain/discomfort and depression/anxiety. For example, the response pattern 11111 corresponds to the state of perfect health with no impairments on any dimension, while the response pattern 11121 indicates no impairment on all dimensions except pain/discomfort, on which slight pain was reported

The utility index at baseline was 0.83 (95% confidence interval: [0.82, 0.85]), 0.87 (95%CI: [0.85, 0.88]) mid-treatment, and significantly rose to 0.90 (95%CI: [0.89, 0.92]; *p* < 0.001) after treatment (Table 4). The EQ-VAS score at baseline was 67.1 (95%CI: [65.6, 68.6]), 70.8 (95%CI: [69.5, 72.1]) mid-treatment, and significantly improved to 79.4 (95%CI: [78.1, 80.6]; *p* < 0.001). A similar recovery in HRQoL was observed among most sub-groups. This result was confirmed after adjustment for participant characteristics (Table 5). Utility indexes significantly improved (β_UI−CP_ = 0.07; 95%CI: [0.03, 0.10]; *p* < 0.001) between intensive and continuation phases and post-treatment (β_UI−EOT_ = 0.16; 95%CI: [0.13, 0.20]; *p* < 0.001). EQ-VAS scores concordantly improved by the continuation phase (β_VAS−CP_ = 4.3; 95%CI: [2.3, 6.3]; *p* < 0.001) and after treatment (β_VAS−EOT_ = 13.0 (95%CI: [10.9, 15.0]; *p* < 0.001). Both changes in the composite indicators surpass the respective MCID thresholds of 0.07 and 9.8.

**Table 4:** EQ-5D-5L utility indexes & EQ-VAS by participant characteristics and stage of treatment

	Utility index							EQ-VAS score							
	*Intensive phase*		*Continuation phase*		*End of treatment*		*p*-value§	*Intensive phase*		*Continuation phase*		*End of treatment*		*p*-value§	
	Mean	95%CI	Mean	95%CI	Mean	95%CI		Mean	95%CI	Mean	95%CI	Mean	95%CI		
**Total**	83	[0.82, 0.85]	0.87	[0.85, 0.88]	0.90	[0.89, 0.92]	< 0.001	67.1	[65.6, 68.6]	70.8	[69.5, 72.1]	79.4	[78.1, 80.6]	< 0.001	
**DEMOGRAPHICS**															
Sex															
Female	0.85	[0.84, 0.87]	0.88	[0.87, 0.90]	0.92	[0.90, 0.93]	< 0.001	68.1	[66.4, 69.9]	71.3	[69.8, 72.8]	79.6	[78.2, 81.0]	0.010	
Male	0.77	[0.73, 0.81]	0.82	[0.79, 0.86]	0.86	[0.82, 0.90]		63.9	[60.8, 66.9]	69.4	[66.6, 72.2]	78.6	[76.0, 81.2]		
*p-value¶*		*< 0.001*		*0.011*		*0.020*			*0.010*		*0.261*		*0.447*		
Age															
<35 years	0.88	[0.86, 0.90]	0.94	[0.92, 0.95]	0.97	[0.95, 0.98]	< 0.001	73.6	[70.8, 76.3]	80.0	[77.8, 82.2]	87.8	[86.0, 89.6]	< 0.001	
35–44 years	0.81	[0.77, 0.86]	0.87	[0.84, 0.90]	0.94	[0.91, 0.96]		65.8	[62.3, 69.4]	72.1	[68.8, 75.3]	79.5	[76.9, 82.1]		
45–54 years	0.84	[0.80, 0.87]	0.86	[0.82, 0.90]	0.89	[0.86, 0.93]		67.7	[64.2, 71.2]	69.2	[66.3, 72.2]	79.6	[76.8, 82.4]		
55–64 years	0.82	[0.78, 0.85]	0.83	[0.80, 0.86]	0.87	[0.84, 0.90]		65.0	[61.8, 68.2]	66.8	[64.1, 69.6]	75.4	[72.7, 78.0]		
65 + years	0.82	[0.77, 0.87]	0.84	[0.79, 0.88]	0.85	[0.79, 0.90]		62.3	[58.6, 66.0]	65.1	[61.8, 68.5]	73.8	[70.4, 77.1]		
*p-value¥*		*0.396*		*< 0.001*		*< 0.001*			*< 0.001*		*< 0.001*		*< 0.001*		
Province															
Ha Noi	0.83	[0.76, 0.89]	0.81	[0.75, 0.86]	0.84	[0.77, 0.90]	< 0.001	71.5	[66.7, 76.3]	70.5	[65.5, 75.5]	76.3	[71.7, 80.8]	0.006	
Hai Phong	0.86	[0.81, 0.91]	0.89	[0.85, 0.93]	0.91	[0.87, 0.95]		69.9	[65.6, 74.1]	68.9	[64.9, 72.8]	76.7	[72.8, 80.6]		
Ho Chi Minh City	0.83	[0.81, 0.85]	0.87	[0.85, 0.89]	0.91	[0.89, 0.93]		65.7	[64.0, 67.5]	70.8	[69.3, 72.4]	80.0	[78.6, 81.3]		
Da Nang	0.94	[0.89, 0.98]	0.92	[0.87, 0.98]	0.94	[0.89, 0.99]		75.5	[68.0, 83.0]	78.9	[74.4, 83.4]	85.4	[80.8, 89.9]		
*p-value¥*		*0.006*		*0.018*		*0.231*			*0.008*		*0.081*		*0.081*		
**CLINICAL CHARACTERISTICS**															
Diagnosis (*N* = 583)															
Bacteriologically confirmed	0.87	[0.79, 0.95]	0.89	[0.83, 0.94]	0.90	[0.85, 0.95]	0.198	73.8	[68.4, 79.2]	73.9	[68.5, 79.4]	80.1	[74.7, 85.6]	0.018	
Clinically diagnosed	0.83	[0.81, 0.85]	0.87	[0.85, 0.88]	0.90	[0.89, 0.92]		66.6	[65.0, 68.2]	70.6	[69.2, 71.9]	79.3	[78.0, 80.6]		
*p-value¶*		*0.044*		*0.608*		*0.685*			*0.037*		*0.146*		*0.583*		
TB site (*N* = 583)															
Pulmonary TB	0.83	[0.82, 0.85]	0.87	[0.85, 0.88]	0.90	[0.89, 0.92]	0.138	67.1	[65.6, 68.6]	70.8	[69.4, 72.1]	79.4	[78.1, 80.6]	0.302	
Extrapulmonary TB	0.77	[0.41, 1.13]	0.80	[0.54, 1.05]	0.81	[0.65, 0.96]		65.0	[50.9, 79.1]	70.0	[58.2, 81.8]	75.9	[64.8, 87.0]		
*p-value¶*		*0.662*		*0.540*		*0.014*			*0.686*		*0.756*		*0.296*		
Treatment category (*N* = 583)															
New	0.85	[0.83, 0.87]	0.87	[0.86, 0.89]	0.91	[0.89, 0.92]	< 0.001	67.6	[66.0, 69.3]	71.2	[69.7, 72.7]	79.4	[78.1, 80.8]	0.037	
Retreatment	0.75	[0.71, 0.80]	0.84	[0.81, 0.87]	0.89	[0.84, 0.93]		64.5	[61.0, 68.0]	69.0	[65.9, 72.0]	78.9	[76.1, 81.7]		
*p-value¶*		*< 0.001*		*0.001*		*0.079*			*0.107*		*0.166*		*0.545*		
Treatment outcome (*N* = 531)															
Treatment success	0.83	[0.81, 0.85]	0.86	[0.85, 0.88]	0.90	[0.89, 0.92]	0.417	67.3	[65.7, 68.9]	71.4	[70.0, 72.8]	79.9	[78.6, 81.2]	0.072	
Unfavorable outcome*	0.84	[0.76, 0.92]	0.92	[0.86, 0.97]	0.91	[0.85, 0.97]		65.4	[59.4, 71.5]	69.2	[61.9, 76.5]	75.2	[69.2, 81.2]		
*p-value¶*		*0.787*		*0.149*		*0.707*			*0.483*		*0.473*		*0.092*		
**HEALTH ACCESS**															
Care pathway															
NTP	0.82	[0.80, 0.84]	0.86	[0.84, 0.88]	0.90	[0.89, 0.92]	0.005	65.5	[63.7, 67.3]	70.5	[68.9, 72.1]	79.5	[78.0, 80.9]	< 0.001	
ACF	0.87	[0.84, 0.91]	0.88	[0.84, 0.91]	0.88	[0.84, 0.93]		70.4	[67.1, 73.7]	69.6	[66.5, 72.6]	77.7	[74.8, 80.7]		
PPM	0.88	[0.83, 0.92]	0.90	[0.86, 0.94]	0.94	[0.91, 0.97]		73.9	[69.2, 78.7]	76.3	[71.8, 80.7]	82.0	[78.1, 85.9]		
*p-value¥*		*0.003*		*0.181*		*0.288*			*0.001*		*0.023*		*0.224*		
Diagnostic delay (*N* = 515)															
1–3 weeks	0.90	[0.88, 0.93]	0.91	[0.88, 0.95]	0.95	[0.93, 0.97]	< 0.001	70.8	[67.0, 74.5]	74.2	[70.7, 77.7]	82.6	[79.6, 85.7]	< 0.001	
4–5 weeks	0.86	[0.82, 0.90]	0.89	[0.85, 0.92]	0.91	[0.87, 0.95]		71.3	[67.5, 75.1]	73.2	[69.7, 76.8]	79.4	[76.1, 82.8]		
6–10 weeks	0.83	[0.80, 0.87]	0.88	[0.85, 0.90]	0.94	[0.92, 0.96]		67.0	[63.4, 70.5]	71.0	[67.7, 74.2]	81.1	[78.7, 83.5]		
11–21 weeks	0.77	[0.72, 0.81]	0.84	[0.81, 0.88]	0.86	[0.82, 0.90]		62.8	[59.3, 66.3]	68.5	[65.5, 71.5]	77.1	[74.0, 80.1]		
22 + weeks	0.82	[0.77, 0.86]	0.84	[0.80, 0.88]	0.88	[0.84, 0.92]		64.1	[60.7, 67.5]	68.0	[65.1, 70.9]	76.0	[72.8, 79.1]		
*p-value¥*		*< 0.001*		*0.013*		*0.003*			*0.002*		*0.016*		*0.010*		
Health-seeking															
1–2 attempts	0.86	[0.81, 0.91]	0.84	[0.80, 0.89]	0.89	[0.84, 0.93]	< 0.001	69.1	[64.9, 73.3]	70.4	[66.9, 73.9]	79.3	[75.8, 82.8]	0.017	
3–4 attempts	0.86	[0.82, 0.89]	0.89	[0.87, 0.92]	0.93	[0.90, 0.95]		69.1	[65.9, 72.3]	72.7	[70.0, 75.4]	80.1	[77.7, 82.4]		
5–6 attempts	0.85	[0.81, 0.88]	0.88	[0.84, 0.91]	0.91	[0.87, 0.94]		68.2	[64.6, 71.8]	72.2	[69.1, 75.3]	79.0	[76.2, 81.9]		
7–10 attempts	0.84	[0.81, 0.88]	0.88	[0.85, 0.91]	0.91	[0.88, 0.94]		66.5	[63.3, 69.6]	69.3	[65.9, 72.7]	79.2	[76.3, 82.0]		
11 + attempts	0.77	[0.73, 0.81]	0.83	[0.79, 0.86]	0.87	[0.84, 0.91]		63.1	[60.2, 66.1]	68.9	[66.4, 71.5]	79.1	[76.3, 81.8]		
*p-value¥*		*< 0.001*		*0.037*		*0.067*			*0.017*		*0.208*		*0.998*		
Social Health Insurance															
No	0.83	[0.79, 0.86]	0.86	[0.83, 0.90]	0.92	[0.89, 0.95]	0.823	64.6	[61.3, 67.9]	71.5	[68.6, 74.3]	79.7	[77.2, 82.1]	0.516	
Yes	0.84	[0.82, 0.86]	0.87	[0.85, 0.88]	0.90	[0.88, 0.92]		67.9	[66.2, 69.5]	70.6	[69.1, 72.1]	79.3	[77.9, 80.7]		
*p-value¶*		*0.554*		*0.857*		*0.389*			*0.099*		*0.606*		*0.989*		
**SOCIOECONOMIC CHARACTERISTICS**															
Education level															
Not literate	0.70	[0.58, 0.81]	0.72	[0.58, 0.86]	0.73	[0.55, 0.91]	< 0.001	50.0	[40.6, 59.4]	66.0	[58.7, 73.3]	73.4	[66.3, 80.6]	< 0.001	
Primary school	0.82	[0.79, 0.84]	0.87	[0.84, 0.89]	0.89	[0.87, 0.92]		63.9	[61.6, 66.1]	68.0	[65.8, 70.1]	77.4	[75.2, 79.5]		
Secondary school	0.85	[0.81, 0.88]	0.86	[0.83, 0.89]	0.90	[0.87, 0.93]		67.9	[64.9, 71.0]	69.6	[66.9, 72.2]	78.3	[75.9, 80.6]		
High school	0.85	[0.82, 0.88]	0.88	[0.85, 0.91]	0.92	[0.90, 0.95]		71.9	[68.4, 75.3]	73.6	[70.3, 76.9]	81.2	[78.4, 84.0]		
University/Post-graduate	0.88	[0.85, 0.91]	0.91	[0.89, 0.93]	0.96	[0.93, 0.98]		73.1	[69.7, 76.4]	78.5	[75.6, 81.4]	86.2	[83.7, 88.7]		
*p-value¥*		*0.003*		*0.160*		*0.003*			*< 0.001*		*< 0.001*		*< 0.001*		
Education length															
0–4 years	0.78	[0.73, 0.83]	0.83	[0.79, 0.88]	0.86	[0.81, 0.91]	0.002	59.8	[56.2, 63.4]	67.6	[64.5, 70.7]	77.4	[74.3, 80.5]	< 0.001	
5–6 years	0.81	[0.76, 0.85]	0.85	[0.81, 0.89]	0.87	[0.83, 0.92]		63.8	[60.2, 67.3]	67.1	[63.6, 70.6]	76.1	[72.8, 79.3]		
7–8 years	0.84	[0.80, 0.89]	0.86	[0.82, 0.89]	0.88	[0.84, 0.92]		65.0	[61.2, 68.9]	66.2	[62.9, 69.5]	74.8	[71.6, 77.9]		
9–11 years	0.86	[0.83, 0.90]	0.89	[0.86, 0.91]	0.92	[0.90, 0.95]		69.6	[66.4, 72.9]	71.4	[68.6, 74.2]	79.2	[76.4, 82.0]		
12 + years	0.86	[0.84, 0.88]	0.89	[0.87, 0.91]	0.95	[0.93, 0.96]		73.2	[70.6, 75.8]	77.3	[75.1, 79.6]	85.4	[83.6, 87.2]		
*p-value¥*		*0.112*		*0.511*		*0.008*			*< 0.001*		*< 0.001*		*< 0.001*		
Head of household															
No	0.82	[0.80, 0.85]	0.87	[0.85, 0.90]	0.91	[0.89, 0.94]	0.802	67.8	[65.5, 70.0]	72.9	[70.9, 74.9]	80.9	[79.2, 82.7]	0.003	
Yes	0.84	[0.82, 0.86]	0.86	[0.84, 0.88]	0.90	[0.87, 0.92]		66.6	[64.6, 68.7]	69.2	[67.4, 71.0]	78.2	[76.5, 79.9]		
*p*-value¶		*0.151*		*0.551*		*0.164*			*0.517*		*0.009*		*0.057*		
Household size															
0–4 years	0.85	[0.80, 0.91]	0.84	[0.79, 0.90]	0.87	[0.80, 0.94]	0.186	66.8	[61.2, 72.3]	67.8	[61.6, 74.1]	77.9	[72.7, 83.2]	0.026	
5–6 years	0.79	[0.74, 0.84]	0.83	[0.78, 0.88]	0.86	[0.80, 0.91]		64.8	[60.7, 68.9]	67.5	[64.1, 70.9]	75.6	[72.2, 79.1]		
7–8 years	0.83	[0.79, 0.87]	0.87	[0.83, 0.90]	0.93	[0.91, 0.96]		66.3	[63.0, 69.7]	69.8	[66.9, 72.8]	81.6	[79.0, 84.2]		
9–11 years	0.85	[0.82, 0.88]	0.88	[0.86, 0.91]	0.92	[0.89, 0.94]		68.3	[65.3, 71.3]	72.2	[69.7, 74.8]	80.4	[77.8, 83.0]		
12 + years	0.84	[0.82, 0.87]	0.88	[0.86, 0.90]	0.91	[0.88, 0.93]		67.9	[65.2, 70.5]	72.5	[70.2, 74.9]	79.4	[77.4, 81.3]		
*p-value¥*		*0.594*		*0.418*		*0.354*			*0.832*		*0.114*		*0.067*		
Primary earner															
No	0.84	[0.81, 0.86]	0.87	[0.85, 0.89]	0.89	[0.87, 0.92]	0.376	67.1	[65.0, 69.1]	70.9	[69.1, 72.7]	79.0	[77.3, 80.6]	0.457	
Yes	0.83	[0.81, 0.86]	0.86	[0.84, 0.88]	0.92	[0.90, 0.94]		67.2	[64.9, 69.4]	70.7	[68.6, 72.7]	79.9	[78.0, 81.7]		
*p-value¶*		*0.349*		*0.202*		*0.434*			*0.675*		*0.987*		*0.395*		
Employment															
Unemployed	0.82	[0.78, 0.86]	0.85	[0.82, 0.88]	0.86	[0.83, 0.90]	< 0.001	66.0	[63.1, 69.0]	68.0	[65.5, 70.6]	77.1	[74.8, 79.5]	< 0.001	
Formally employed	0.87	[0.84, 0.90]	0.91	[0.88, 0.94]	0.95	[0.92, 0.98]		73.1	[69.7, 76.6]	76.8	[73.6, 80.0]	84.6	[81.2, 88.0]		
Informally employed	0.84	[0.81, 0.86]	0.87	[0.85, 0.89]	0.92	[0.90, 0.94]		66.5	[64.2, 68.7]	72.3	[70.4, 74.2]	80.4	[78.7, 82.2]		
Don’t know/No answer	0.82	[0.77, 0.87]	0.84	[0.80, 0.89]	0.90	[0.87, 0.93]		65.8	[61.7, 69.8]	65.5	[61.4, 69.6]	75.2	[71.7, 78.6]		
*p-value¥*		*0.750*		*0.215*		*< 0.001*			*0.014*		*< 0.001*		*< 0.001*		
Pre-TB monthly income															
USD 0–24	0.82	[0.77, 0.86]	0.84	[0.80, 0.89]	0.87	[0.83, 0.91]	0.012	63.8	[60.2, 67.3]	66.4	[63.2, 69.6]	75.6	[72.6, 78.7]	< 0.001	
USD 25–169	0.83	[0.79, 0.87]	0.86	[0.82, 0.89]	0.87	[0.83, 0.91]		67.6	[64.2, 71.0]	70.3	[67.2, 73.4]	78.6	[75.9, 81.3]		
USD 170–259	0.82	[0.78, 0.86]	0.85	[0.82, 0.89]	0.90	[0.87, 0.94]		66.2	[62.9, 69.5]	70.2	[67.2, 73.1]	80.2	[77.5, 82.9]		
USD 260–389	0.85	[0.82, 0.88]	0.91	[0.89, 0.93]	0.94	[0.92, 0.97]		68.2	[64.9, 71.5]	74.1	[71.2, 77.0]	80.9	[78.2, 83.7]		
USD 390+	0.86	[0.82, 0.89]	0.88	[0.85, 0.91]	0.93	[0.90, 0.96]		70.1	[66.8, 73.4]	73.4	[70.6, 76.2]	81.6	[79.0, 84.2]		
*p-value¥*		*0.911*		*0.234*		*0.006*			*0.070*		*0.002*		*0.048*		
**SOCIOECONOMIC IMPACT OF TB**															
Monthly income decline															
No decline	0.87	[0.85, 0.89]	0.88	[0.86, 0.91]	0.92	[0.89, 0.94]	< 0.001	68.5	[66.1, 70.8]	72.1	[69.9, 74.3]	80.7	[78.9, 82.6]	0.062	
USD 1-100	0.80	[0.75, 0.85]	0.85	[0.80, 0.89]	0.87	[0.82, 0.92]		66.1	[62.1, 70.0]	68.4	[64.9, 71.9]	78.3	[74.9, 81.7]		
USD 101–250	0.83	[0.79, 0.87]	0.85	[0.81, 0.89]	0.89	[0.85, 0.92]		67.4	[63.8, 71.1]	68.9	[65.4, 72.4]	76.7	[73.7, 79.7]		
USD 251–400	0.79	[0.75, 0.83]	0.88	[0.85, 0.90]	0.92	[0.89, 0.95]		64.6	[60.6, 68.5]	72.0	[69.1, 74.9]	79.7	[76.3, 83.0]		
USD 401+	0.82	[0.77, 0.88]	0.85	[0.81, 0.90]	0.91	[0.86, 0.95]		67.0	[62.5, 71.4]	70.3	[66.7, 74.0]	79.4	[75.7, 83.0]		
*p-value¥*		*< 0.001*		*0.022*		*0.226*			*0.554*		*0.408*		*0.157*		
Job loss															
No	0.85	[0.83, 0.87]	0.88	[0.86, 0.89]	0.90	[0.89, 0.92]	< 0.001	68.7	[67.1, 70.3]	71.0	[69.5, 72.6]	79.7	[78.3, 81.0]	< 0.001	
Yes	0.76	[0.72, 0.81]	0.83	[0.80, 0.87]	0.90	[0.87, 0.93]		60.6	[57.0, 64.2]	69.8	[67.2, 72.4]	78.0	[75.0, 81.0]		
*p-value¶*		*< 0.001*		*0.001*		*0.552*			*<0.001*		*0.393*		*0.349*		
Borrow or receive cash															
No	0.85	[0.83, 0.87]	0.88	[0.86, 0.89]	0.91	[0.89, 0.93]	< 0.001	68.3	[66.5, 70.0]	71.5	[69.9, 73.0]	80.1	[78.7, 81.5]	< 0.001	
Yes	0.79	[0.76, 0.83]	0.84	[0.81, 0.87]	0.89	[0.86, 0.92]		64.1	[61.1, 67.0]	69.1	[66.5, 71.6]	77.4	[74.9, 79.9]		
*p-value¶*		*0.015*		*0.014*		*0.015*			*0.013*		*0.143*		*0.057*		
Sell assets															
No	0.84	[0.82, 0.86]	0.87	[0.86, 0.89]	0.91	[0.89, 0.92]	< 0.001	67.7	[66.2, 69.2]	71.3	[69.9, 72.6]	79.6	[78.3, 80.9]	< 0.001	
Yes	0.75	[0.67, 0.84]	0.77	[0.69, 0.86]	0.88	[0.80, 0.95]		56.7	[49.8, 63.6]	61.8	[54.5, 69.1]	74.8	[69.9, 79.8]		
*p-value¶*		*0.032*		*0.013*		*0.068*			*0.002*		*0.006*		*0.028*		

Notes: TB = Tuberculosis; ACF = Active Case Finding; PPM = Public-Private-Mix; § *P*-values in these columns denote statistical differences in longitudinal changes in utility index and EQ-VAS for each sub-group using RM-ANOVA or Friedman’s test; ¶ *P*-values in these rows denote statistical differences in utility index and EQ-VAS within sub-groups for each treatment stage using Mann-Whitney tests; ¥ *P*-values in these rows denote statistical differences in utility index and EQ-VAS within sub-groups for each treatment stage using Kruskal-Wallis tests; * Unfavorable treatment outcomes include treatment failure, loss to follow-up, transfer out and death

**Table 5:** Associations between EQ-5D-5L utility indexes & EQ-VAS and participant characteristics

	Utility index			EQ-VAS score		
	Coefficient (β_UI_)	95%CI	*p*-value^§^	Coefficient (β_VAS_)	95%CI	*p*-value^§^
**PRIMARY EXPOSURES**						
Treatment stage						
Intensive phase	Ref			Ref		
Continuation phase	0.07	[0.04, 0.09]	< 0.001	4.3	[1.9, 6.6]	< 0.001
End of treatment	0.16	[0.14, 0.19]	< 0.001	13.0	[8.9, 17.0]	< 0.001
Care pathway						
NTP	Ref			Ref		
ACF	0.07	[0.02, 0.13]	0.008	6.4	[5.2, 7.6]	< 0.001
PPM	0.03	[-0.04, 0.10]	0.472	1.5	[-2.5, 5.5]	0.461
**DEMOGRAPHICS**						
Sex						
Female	Ref			Ref		
Male	0.08	[0.05, 0.11]	< 0.001	2.5	[1.9, 3.1]	< 0.001
Age						
<35 years	Ref			Ref		
35–44 years	-0.11	[-0.12, -0.10]	< 0.001	-9.4	[-11.3, -7.5]	< 0.001
45–54 years	-0.13	[-0.15, -0.10]	< 0.001	-8.3	[-9.9, -6.7]	< 0.001
55–64 years	-0.15	[-0.18, -0.12]	< 0.001	-11.5	[-16.8, -6.3]	< 0.001
65 + years	-0.18	[-0.21, -0.14]	< 0.001	-14.2	[-16.1, -12.3]	< 0.001
**CLINICAL CHARACTERISTICS**						
Diagnosis						
Bacteriologically confirmed	Ref			Ref		
Clinically diagnosed	-0.04	[-0.11, 0.04]	0.369	-3.8	[-7.6, 0.0]	0.048
TB site						
Pulmonary TB	Ref			Ref		
Extrapulmonary TB	-0.14	[-0.43, 0.14]	0.315	-8.3	[-14.4, -2.2]	0.008
Treatment category						
New	Ref			Ref		
Retreatment	-0.07	[-0.08, -0.05]	< 0.001	-1.6	[-3.3, 0.1]	0.059
Treatment outcome						
Treatment success	Ref			Ref		
Unfavorable outcome*	0.01	[-0.07, 0.08]	0.868	-4.7	[-6.8, -2.7]	< 0.001
**HEALTH ACCESS**						
Diagnostic delay						
1–3 weeks	Ref			Ref		
4–5 weeks	-0.05	[-0.08, -0.03]	< 0.001	-3.2	[-3.8, -2.6]	< 0.001
6–10 weeks	-0.06	[-0.12, 0.00]	0.049	-3.7	[-5.1, -2.3]	< 0.001
11–21 weeks	-0.11	[-0.14, -0.09]	< 0.001	-5.3	[-6.6, -4.0]	< 0.001
22 + weeks	-0.09	[-0.12, -0.07]	< 0.001	-5.8	[-7.5, -4.0]	< 0.001
Health-seeking						
1–2 attempts	Ref			Ref		
3–4 attempts	0.05	[-0.05, 0.15]	0.344	0.0	[-5.9, 5.8]	0.992
5–6 attempts	0.06	[-0.02, 0.14]	0.141	1.1	[-4.3, 6.5]	0.687
7–10 attempts	0.09	[0.02, 0.15]	0.008	2.0	[-2.8, 6.8]	0.413
11 + attempts	0.00	[-0.09, 0.10]	0.916	-0.5	[-4.4, 3.4]	0.804
Social Health Insurance						
No	Ref			Ref		
Yes	0.02	[-0.01, 0.05]	0.310	2.3	[1.8, 2.7]	< 0.001
**SOCIOECONOMIC CHARACTERISTICS**						
Education level						
Not literate	Ref			Ref		
Primary school	0.14	[0.11, 0.18]	< 0.001	6.8	[4.9, 8.6]	< 0.001
Secondary school	0.14	[0.10, 0.18]	< 0.001	6.5	[3.3, 9.7]	< 0.001
High school	0.12	[-0.02, 0.25]	0.093	0.1	[-5.3, 5.6]	0.964
University/Post-graduate	0.14	[-0.01, 0.30]	0.074	-1.2	[-5.6, 3.1]	0.575
Education length						
0–4 years	Ref			Ref		
5–6 years	-0.04	[-0.06, -0.01]	0.001	-1.8	[-2.7, -1.0]	< 0.001
7–8 years	-0.01	[-0.04, 0.02]	0.544	-2.8	[-6.1, 0.5]	0.101
9–11 years	-0.02	[-0.05, 0.01]	0.246	0.4	[-1.3, 2.1]	0.628
12 + years	-0.02	[-0.19, 0.14]	0.762	9.5	[3.9, 15.0]	0.001
Head of household						
No	Ref			Ref		
Yes	0.03	[-0.01, 0.07]	0.124	0.9	[-0.6, 2.4]	0.233
Household size						
0–4 years	Ref			Ref		
5–6 years	-0.03	[-0.08, 0.01]	0.143	1.2	[0.0, 2.3]	0.044
7–8 years	0.01	[-0.07, 0.09]	0.794	2.3	[-0.2, 4.7]	0.075
9–11 years	0.03	[-0.04, 0.10]	0.380	2.5	[1.2, 3.7]	< 0.001
12 + years	0.04	[-0.04, 0.12]	0.370	4.0	[2.4, 5.6]	< 0.001
Primary earner						
No	Ref			Ref		
Yes	-0.04	[-0.05, -0.02]	< 0.001	-0.8	[-1.8, 0.1]	0.090
Employment						
Unemployed	Ref			Ref		
Formally employed	0.05	[0.00, 0.10]	0.040	1.1	[-1.5, 3.6]	0.416
Informally employed	0.04	[0.01, 0.08]	0.020	1.4	[0.1, 2.7]	0.039
Don’t know/No answer	0.02	[-0.02, 0.06]	0.447	-0.6	[-1.6, 0.4]	0.252
Pre-TB monthly income						
USD 0–24	Ref			Ref		
USD 25–169	0.03	[-0.03, 0.09]	0.365	6.0	[3.4, 8.5]	< 0.001
USD 170–259	0.03	[-0.03, 0.09]	0.357	4.6	[3.9, 5.2]	< 0.001
USD 260–389	0.08	[0.03, 0.13]	0.001	4.2	[1.8, 6.6]	0.001
USD 390+	0.04	[-0.03, 0.11]	0.300	6.5	[2.1, 10.9]	0.004
**SOCIOECONOMIC IMPACT OF TB**						
Monthly income decline						
No decline	Ref			Ref		
USD 1-100	-0.07	[-0.12, -0.02]	0.004	-2.2	[-2.8, -1.7]	< 0.001
USD 101–250	-0.07	[-0.10, -0.03]	< 0.001	-2.9	[-4.3, -1.4]	< 0.001
USD 251–400	-0.08	[-0.11, -0.06]	< 0.001	-1.6	[-2.4, -0.8]	< 0.001
USD 401+	-0.07	[-0.13, -0.01]	0.017	-4.2	[-7.2, -1.3]	0.005
Job loss						
No	Ref			Ref		
Yes	-0.04	[-0.10, 0.02]	0.178	-2.5	[-4.7, -0.2]	0.032
Borrow or receive cash						
No	Ref			Ref		
Yes	-0.04	[-0.07, 0.00]	0.027	-0.8	[-2.8, 1.3]	0.480
Sell assets						
No	Ref			Ref		
Yes	-0.03	[-0.07, 0.02]	0.249	-4.8	[-7.6, -2.1]	0.001

Notes: Mixed-effect, multivariate Tobit regression with province as the random effect in a complete-case analysis (*N* = 468) with an interaction term between care pathway and treatment stage; § Wald test; * Unfavorable treatment outcomes include treatment failure, loss to follow-up, transfer out and death

**Fig. 3:**
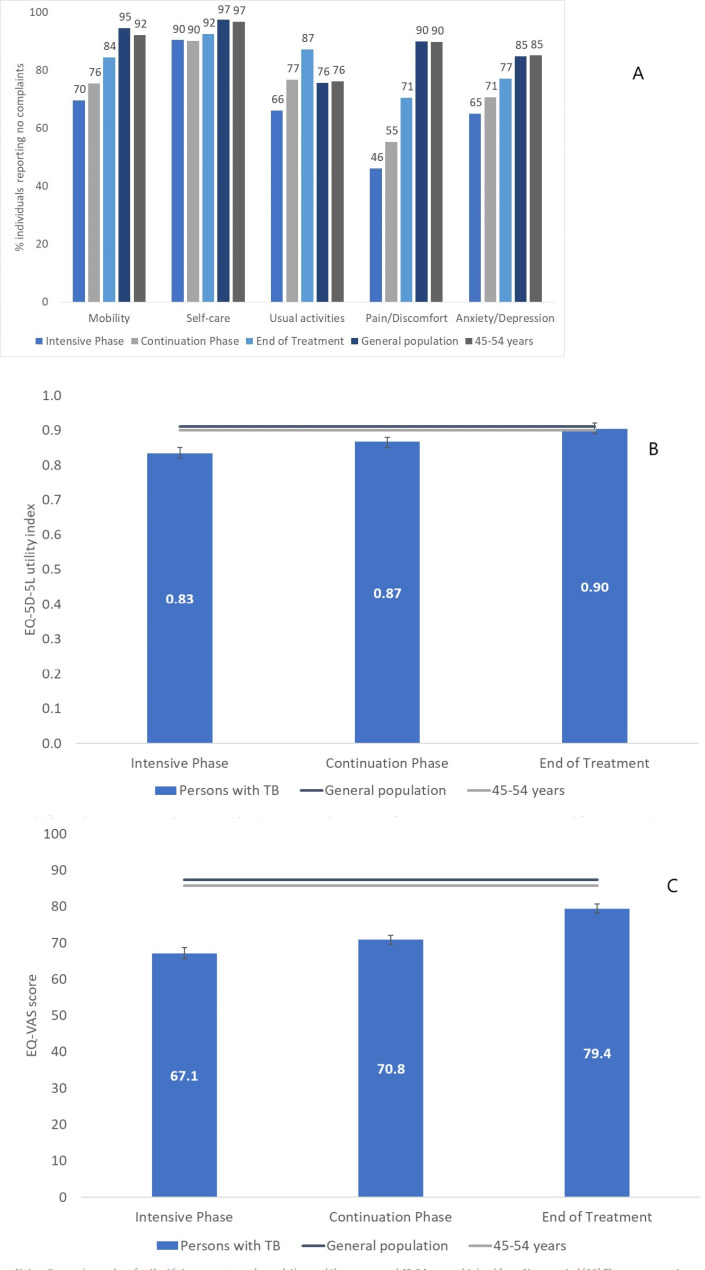
(**A**) EQ-5D-5L dimensions, (**B**) utility index and (**C**) EQ-VAS score by stage of treatment with comparison values from the Vietnamese general population and sub-group of persons aged 45–54 years. Notes: Comparision values for the Vietnamese general population and the group aged 45-54 years obtained from Nguyen et al. [16]. The age segment comparator was chosen based on the median age of the sample

Comparing the EQ-5D-5L dimensions to those of Viet Nam’s general population and the sub-group of persons aged 45–54 years, four of five dimensions remained significantly below both comparators (*p* < 0.001) by the end of treatment with *usual activities* as the only exception (Fig. 3a). The utility indexes (Fig. 3b) and EQ-VAS scores (Fig. 3c) were significantly lower at the beginning of treatment. Post-treatment, the utility indexes were similar to the comparator levels, but the self-rated EQ-VAS score remained significantly below the population averages.

### Differences in HRQoL by care pathway

At baseline, utility indexes for the ACF and PPM pathways were 0.87 (95%CI: [0.84, 0.91]) and 0.88 (95%CI: [0.83, 0.92]), respectively, compared to 0.82 (95%CI: [0.80, 0.84]) in the NTP cohort (Table 4). Similarly, EQ-VAS scores for ACF (70.4; 95%CI: [67.1; 73.7]) and PPM (73.9; 95%CI: [69.2; 78.7]) were higher than the score of the NTP pathway (65.5; 95%CI: [63.7; 67.3]). These differences were significant for both utility indexes (*p* = 0.003) and EQ-VAS (*p* = 0.001). The pairwise comparison of utility indexes showed that only the ACF pathway was significantly higher than the NTP pathway (*p* = 0.003), while the EQ-VAS scores of both ACF (*p* = 0.015) and PPM (*p* = 0.003) pathways were significantly higher (Supplementary Information, Table S3). Post-treatment, there were no significant differences between the three cohorts in utility index (*p* = 0.288) and EQ-VAS score (*p* = 0.224). Results from the multivariate analysis (Table 5) were similar, as adjusted coefficients of utility indexes for the ACF and PPM pathways were β_UI−ACF_ = 0.07 (95%CI: [0.02, 0.13]; *p* = 0.008) and β_UI−PPM_ = 0.03 (95%CI: [-0.04, 0.09]; *p* = 0.472), respectively. Concordantly, respective adjusted coefficients of the EQ-VAS score were β_VAS−ACF_ = 6.4 (95%CI: [5.2, 7.6]; *p* < 0.001) and β_VAS−PPM_ = 1.5 (95%CI: [-2.5, 5.5]; *p* = 0.461).

### Psychometric properties of the EQ-5D-5L tool among persons with TB in Viet Nam

Internal consistency was reliable with Cronbach’s alpha ranging from 0.75 to 0.84 across treatment stages. We detected moderate to high convergent validity between the utility indexes and EQ-VAS scores at baseline (*ρ*_0_ = 0.4679; *p* < 0.001), mid-treatment (*ρ*_0_ = 0.5110; *p* < 0.001) and post-treatment (*ρ*_0_ = 0.5651; *p* < 0.001). In terms of known-groups validity, utility indexes and EQ-VAS scores at baseline (Table 4) were significantly higher among women (Utility index: *p* < 0.001; EQ-VAS score: *p* = 0.010) as well as persons with shorter diagnostic delay (*p* < 0.001; *p* = 0.002), fewer health-seeking attempts (*p* < 0.001; *p* = 0.002) and higher levels of education (*p* = 0.003; *p* < 0.001). Job loss (both *p* < 0.001), borrowing money (*p* = 0.015; *p* = 0.013) and selling assets (*p* = 0.032; *p* = 0.002) was associated with lower HRQoL. Post-treatment, HRQoL was significantly higher among younger and formally employed persons (all *p* < 0.001) and those with higher pre-TB incomes (*p* = 0.006; *p* = 0.048).

## Discussion

Our study found that TB has a substantial negative impact on HRQoL of affected individuals in Viet Nam. Over the course of treatment, participants reported a significant recovery in HRQoL. However, this recovery remains incomplete on four of five EQ-5D-5L dimensions and EQ-VAS scores when compared to population-level benchmarks. Employing well-established intervention strategies such as ACF may be promising levers to limit the negative impact of TB on HRQoL. Lastly, the EQ-5D-5L is an appropriate tool for measuring HRQoL among persons with TB in Viet Nam.

The harmful effects of TB on health-related quality of life observed on our study are concordant with evidence from other high-burden settings that reported utility indexes of 0.67–0.80 among persons with TB [13, 32, 67]. The utility indexes and EQ-VAS scores were significantly lower than those of the Vietnamese population and age-matched sub-population. About 54% experienced pain and discomfort and 35% suffered from anxiety and depression, which fell in between the results of studies from Pakistan and Nepal [48, 68]. These findings suggest high rates of psychological comorbidities and mental stress from this stigmatized disease as observed on past studies [69, 70]. About one-third of participants reported some degree of impaired mobility and inability to carry out usual activities, which are key contributors to one’s sense of autonomy and independence, and by extension of HRQoL in persons with spinal cord injury and older populations [71, 72]. Linkages between autonomy and independence and TB are poorly understood and represent a key research gap [4, 73]. 

We found that TB treatment improved HRQoL and wellbeing [32, 74]. This improvement was reflected across virtually all measures. The proportion of persons reporting no complaint significantly rose on all but one of the EQ-5D-5L dimensions, the utility indexes and EQ-VAS scores. These composite measures also exceeded the respective MCID’s suggesting that treatment made a marked clinical difference for TB-affected individuals, which was similar to findings reported from South Africa and Nepal [30, 49]. 

Our study also identified areas requiring further interventions post TB treatment [75]. Even though the rate of impairment in each dimension declined, this did not account for the depth of impairment. Specifically, our data showed the largest shift from mild impairment to no impairment, while the proportion of moderate to extreme impairment changed very little. Thus, post-treatment measurements of four out of five EQ-5D-5L dimensions and EQ-VAS showed that life quality in persons with TB remains below that of the general population despite being successfully treated [76–78]. This suggests that TB treatment by itself may remedy light ailments, but that persons with moderate to extreme disability will require support beyond clinical care both during and after treatment.

A key reason for the muted recovery may be due to TB sequalae commonly observed among persons with TB. Post-TB lung disease commonly includes abnormal lung function, bronchiectasis, and increase the risk of lung infections, malignancies, and concomitant heart failure. Found in about half of previously treated persons with TB, these have been associated with a range of predictors such as severe pulmonary TB characterized by lung cavitation and consolidation on chest X-ray, bilateral lung involvement, female gender and behavioral risk factors such as smoking. As a result, it was concluded that these sequelae contribute to increased morbidity and mortality. Moreover, studies have highlighted the chronic, oftentimes undiagnosed nature of these spirometric declines, leading to long-term impairment of health-related quality of life [33, 79–82]. 

Long-term mental illness, psychogenic pain and psychosomatic disorders are also common among TB survivors [83, 84]. Reports of *pain/discomfort* in our study declined from 54 to 29% following treatment but remained far above the rate of 10% in the general population. Similarly, the post-TB proportion of *anxiety/depression* was 23% compared to 15% in Vietnamese society. Hence, there is growing consensus for the need of multidimensional treatment support and post-TB clinical care such as physical and mental health rehabilitation [34, 85, 86]. However, this area remains a vital research and programmatic gap for persons with TB to be able to return to full health and achieve a full restoration in health-related quality of life.

Our findings related to the observed socioeconomic impairments on HRQoL such as loss of employment (utility index, intensive phase: 0.85 versus 0.76; *p* < 0.001) and income (0.87 versus 0.79–0.83; *p* < 0.001) were also aligned with available evidence [87]. It is understood that an episode of TB carries a high risk of catastrophic cost [88]. For this reason WHO has identified eliminating catastrophic costs as one of the three core measures of success in the End TB Strategy [89]. Similarly, the second UN High-Level Meeting on TB clearly calls for “psychosocial, nutritional and socioeconomic support for successful treatment, including to reduce stigma and discrimination.” [90] Seminal studies like the HRESIPT and RATIONS trials have shown that social protection can improve clinical outcomes and defray catastrophic costs [91–94]. It is conceivable that these socio-protective actions will catalyze a faster and more complete recovery in health-related quality of life.

Prior research from Viet Nam found ACF to possess such socio-protective properties with lower risk of catastrophic costs in persons with TB reached through this care pathway [54]. Other studies have shown that ACF can reach persons with TB at an earlier stage of disease progression with lower symptomatic presentation, which has been associated with higher HRQoL [33]. Our study supports this notion, as crude and adjusted analyses exhibited higher utility indexes and EQ-VAS scores in the ACF cohort compared to the NTP cohort. These findings are discordant with results from Nepal, where ACF was neither associated with a higher HRQoL nor a significant reduction in catastrophic costs, which may have been related to the smaller sample size and differences in statistical analysis methods [95]. Our study showed no difference in HRQoL between the PPM and NTP cohorts, which matched the results from our concurrent patient cost survey, on which we did not detect a difference in catastrophic costs in this pairwise comparison [56]. This result may warrant further research to tailor social support and protection among persons with TB taking private sector care.

### Methodological considerations

Our study benefitted from prior utilization and validation of the EQ-5D-5L tool in Viet Nam, which availed benchmarks for the general population and furnished proxy thresholds and a priori known groups for the psychometric evaluation. For meaningful comparisons with these available benchmarks, we reported means rather than medians despite skewness in the data. Another advantage was the ability to mitigate selection bias by combining four longitudinal HRQoL surveys into a more representative dataset, spanning Viet Nam’s three demographic regions and three care pathways. Regarding the latter, our access to a network of PPM providers afforded rare insights into privately treated individuals with TB [96]. 

A weakness was the small PPM cohort from low participation rates over confidentiality concerns among providers and patients. Consecutive, non-randomized recruitment resulted in oversampling of persons with new, bacteriologically-confirmed, pulmonary TB who completed treatment successfully thus limiting generalizability and external validity in specific sub-groups such as persons with extrapulmonary TB. This may be reflected in some unexpected results with respect to known groups. On our study, women with TB had a higher HRQoL, while there was no difference in length of education and level of pre-TB income. The third surveys all took place shortly after treatment completion, so that the long-term effects of treatment on HRQoL remain unclear. Combining dataset from different studies with specific purposes may have introduced bias, but was considered tolerable given the sample size.

## Conclusion

Tuberculosis has a detrimental impact on the health-related quality of life of affected persons in Viet Nam. Successful treatment restores HRQoL for most people with TB, yet there remains a minority with greater depth of impairment who require sustained support to remedy the clinical, socioeconomic and psychosocial sequelae post-TB. Our results support the notion that the current paradigm of focusing on pharmacologic interventions for six months is insufficient. We conclude that multidimensional support during and after treatment is crucial for affected persons to return to their life before TB, and that post-TB care and social protection should be included in national TB guidelines and long-term disability policies.

## Electronic supplementary material

Below is the link to the electronic supplementary material.

Supplementary Material 1

## Data Availability

The data that support the findings of this study are available from the Viet Nam National Lung Hospital/NTP and Provincial Lung Hospitals of Ha Noi, Hai Phong, Da Nang and Ho Chi Minh City. However, restrictions apply to the availability of these data, which include programmatic clinical patient information, and so are not publicly available. Data can be made available from the authors upon reasonable request and with permission of the relevant government authorities listed above.

## Abbreviations

ACF: Active Case Finding
CXR: Chest X-ray
DS-TB: Drug-Susceptible TB
EQ-5D-5L: EuroQol–5-Dimension–5-Level
EQ-VAS: EQ-5D-5L Visual Analog Scale
HCMC: Ho Chi Minh City
HRQoL: Health-Related Quality of Life
MCID: Minimum Clinically Important Difference
NTP: National TB Control Program
PCS: Patient Cost Survey
PPM: Public-PrivateMix
TB: Tuberculosis
WHO: World Health Organization
