# Supplementary material for: A life that’s worth living – measuring health-related quality of life among people treated for tuberculosis in Viet Nam: a longitudinal EQ-5D-5L survey
Source: Health Qual Life Outcomes. 2025 Apr 23;23:43. doi: 10.1186/s12955-025-02369-9 (PMC12016289; doi:10.1186/s12955-025-02369-9)
Supplement: Supplementary file 1 — Supplementary Material 1 [file 12955_2025_2369_MOESM1_ESM.docx]

Supplementary information

# Supplementary results

**Figure S1. Flow diagram for participant recruitment.**

**Table S1. Participant characteristics by care pathway.**

|  | **NTP** | | **ACF** | | **PPM** | |  |
| --- | --- | --- | --- | --- | --- | --- | --- |
|  | N | % | N | % | N | % | p-value^¥^ |
| **Total** | 429 | 73.3 | 106 | 18.1 | 50 | 8.6 |  |
| **DEMOGRAPHICS** |  |  |  |  |  |  |  |
| Sex |  |  |  |  |  |  |  |
| Female | 103 | 24 | 18 | 17 | 17 | 34 | 0.060 |
| Male | 326 | 76 | 88 | 83 | 33 | 66 |  |
| Age |  |  |  |  |  |  |  |
| <35 years | 110 | 25.6 | 3 | 2.8 | 16 | 32 | <0.001 |
| 35-44 years | 83 | 19.4 | 5 | 4.7 | 10 | 20 |  |
| 45-54 years | 85 | 19.8 | 20 | 18.9 | 8 | 16 |  |
| 55-64 years | 104 | 24.2 | 41 | 38.7 | 10 | 20 |  |
| 65+ years | 47 | 11 | 37 | 34.9 | 6 | 12 |  |
| *Participant age (median, IQR)* | *47* | *(34-58)* | *61* | *(54-68)* | *43* | *(28-60)* |  |
| City |  |  |  |  |  |  |  |
| Ha Noi | 41 | 9.6 | 12 | 11.3 | 2 | 4 | <0.001 |
| Hai Phong | 36 | 8.4 | 23 | 21.7 | 17 | 34 |  |
| Ho Chi Minh City | 342 | 79.7 | 61 | 57.6 | 31 | 62 |  |
| Da Nang | 10 | 2.3 | 10 | 9.4 | 0 | 0 |  |
| **CLINICAL CHARACTERISTICS** |  |  |  |  |  |  |  |
| Diagnosis (N=583) |  |  |  |  |  |  |  |
| Bacteriologically confirmed | 26 | 6.1 | 2 | 1.9 | 10 | 20.0 | <0.001 |
| Clinically diagnosed | 401 | 93.9 | 104 | 98.1 | 40 | 80.0 |  |
| TB location (N=583) |  |  |  |  |  |  |  |
| Pulmonary TB | 419 | 98.1 | 106 | 100.0 | 50 | 100.0 | 0.227 |
| Extrapulmonary TB | 8 | 1.9 | 0 | 0.0 | 0 | 0.0 |  |
| Notification category (N=583) |  |  |  |  |  |  |  |
| New | 325 | 76.1 | 106 | 100.0 | 48 | 96.0 | 0.227 |
| Relapse/Retreatment | 102 | 23.9 | 0 | 0.0 | 2 | 4.0 |  |
| Treatment outcome (N=531) |  |  |  |  |  |  |  |
| Treatment success | 378 | 95.2 | 79 | 94.1 | 50 | 100.0 | 0.243 |
| Unfavorable outcomeⱡ | 19 | 4.8 | 5 | 6.0 | 0 | 0.0 |  |
| **HEALTH ACCESS** |  |  |  |  |  |  |  |
| Diagnostic delay (N=515) |  |  |  |  |  |  |  |
| 1-3 weeks | 76 | 18.7 | 6 | 10 | 8 | 16.7 | 0.787 |
| 4-5 weeks | 67 | 16.5 | 12 | 20 | 7 | 14.6 |  |
| 6-10 weeks | 95 | 23.3 | 12 | 20 | 13 | 27.1 |  |
| 11-21 weeks | 88 | 21.6 | 14 | 23.3 | 11 | 22.9 |  |
| 22+ weeks | 81 | 19.9 | 16 | 26.7 | 9 | 18.8 |  |
| *Diagnostic delay (median, IQR)* | *8* | *(4-18)* | *11* | *(5-23)* | *8* | *(5-16)* |  |
| Health-seeking |  |  |  |  |  |  |  |
| 1-2 attempts | 32 | 7.5 | 35 | 33 | 11 | 22 | <0.001 |
| 3-4 attempts | 91 | 21.2 | 44 | 41.5 | 14 | 28 |  |
| 5-6 attempts | 90 | 21 | 14 | 13.2 | 11 | 22 |  |
| 7-10 attempts | 105 | 24.5 | 5 | 4.7 | 10 | 20 |  |
| 11+ attempts | 111 | 25.9 | 8 | 7.6 | 4 | 8 |  |
| *Health-seeking attempts (median, IQR)* | *7* | *(4-11)* | *3* | *(2-5)* | *5* | *(3-7)* |  |
| Social Health Insurance |  |  |  |  |  |  |  |
| No | 107 | 24.9 | 18 | 17 | 8 | 16 | 0.107 |
| Yes | 322 | 75.1 | 88 | 83 | 42 | 84 |  |
| **SOCIOECONOMIC CHARACTERISTICS** |  |  |  |  |  |  |  |
| Education level |  |  |  |  |  |  |  |
| Not literate | 18 | 4.2 | 6 | 5.7 | 1 | 2 | <0.001 |
| Primary school | 163 | 38 | 42 | 39.6 | 8 | 16 |  |
| Secondary school | 111 | 25.9 | 35 | 33 | 10 | 20 |  |
| High school | 85 | 19.8 | 17 | 16 | 9 | 18 |  |
| University/Post-graduate | 52 | 12.1 | 6 | 5.7 | 22 | 44 |  |
| Education length |  |  |  |  |  |  |  |
| 0-4 years | 18 | 4.2 | 6 | 5.7 | 1 | 2 | <0.001 |
| 5-6 years | 163 | 38 | 42 | 39.6 | 8 | 16 |  |
| 7-8 years | 111 | 25.9 | 35 | 33 | 10 | 20 |  |
| 9-11 years | 85 | 19.8 | 17 | 16 | 9 | 18 |  |
| 12+ years | 52 | 12.1 | 6 | 5.7 | 22 | 44 |  |
| *Years of education (median, IQR)* | *8* | *(5-12)* | *7* | *(5-9)* | *12* | *(7-16)* |  |
| Head of household |  |  |  |  |  |  |  |
| No | 199 | 46.4 | 28 | 26.4 | 25 | 50.0 | 0.001 |
| Yes | 230 | 53.6 | 78 | 73.6 | 25 | 50.0 |  |
| Household size |  |  |  |  |  |  |  |
| 0-4 years | 36 | 8.4 | 6 | 5.7 | 2 | 4 | 0.418 |
| 5-6 years | 66 | 15.4 | 18 | 17 | 7 | 14 |  |
| 7-8 years | 85 | 19.8 | 18 | 17 | 11 | 22 |  |
| 9-11 years | 110 | 25.6 | 19 | 17.9 | 13 | 26 |  |
| 12+ years | 132 | 30.8 | 45 | 42.5 | 17 | 34 |  |
| *Household size (median, IQR)* | *4* | *(3-5)* | *4* | *(3-5)* | *4* | *(3-5)* |  |
| Primary earner |  |  |  |  |  |  |  |
| No | 213 | 49.7 | 76 | 71.7 | 32 | 64 | <0.001 |
| Yes | 216 | 50.4 | 30 | 28.3 | 18 | 36 |  |
| Employment |  |  |  |  |  |  |  |
| Unemployed | 103 | 24 | 47 | 44.3 | 17 | 34 | <0.001 |
| Formally employed | 63 | 14.7 | 5 | 4.7 | 9 | 18 |  |
| Informally employed | 212 | 49.4 | 38 | 35.9 | 17 | 34 |  |
| Don't know/No answer | 51 | 11.9 | 16 | 15.1 | 7 | 14 |  |
| Pre-TB monthly income |  |  |  |  |  |  |  |
| USD 0-24 | 76 | 17.7 | 33 | 31.1 | 8 | 16 | <0.001 |
| USD 25-169 | 74 | 17.3 | 35 | 33 | 8 | 16 |  |
| USD 170-259 | 101 | 23.5 | 18 | 17 | 6 | 12 |  |
| USD 260-389 | 92 | 21.5 | 10 | 9.4 | 13 | 26 |  |
| USD 390+ | 86 | 20.1 | 10 | 9.4 | 15 | 30 |  |
| *Pre-TB monthly income (median, IQR)* | *234* | *(85-362)* | *99* | *(0-253)* | *298* | *(128-468)* |  |
| **SOCIOECONOMIC IMPACT OF TB** |  |  |  |  |  |  |  |
| Monthly income decline |  |  |  |  |  |  |  |
| No decline | 167 | 38.9 | 57 | 53.8 | 17 | 34 | 0.003 |
| USD 1-100 | 61 | 14.2 | 25 | 23.6 | 8 | 16 |  |
| USD 101-250 | 73 | 17 | 11 | 10.4 | 9 | 18 |  |
| USD 251-400 | 77 | 18 | 8 | 7.6 | 8 | 16 |  |
| USD 401+ | 51 | 11.9 | 5 | 4.7 | 8 | 16 |  |
| *Loss in monthly income (median, IQR)* | *85* | *(0-266)* | *0* | *(0-94)* | *113* | *(0-298)* |  |
| Job loss |  |  |  |  |  |  |  |
| No | 325 | 75.8 | 101 | 95.3 | 46 | 92 | <0.001 |
| Yes | 104 | 24.2 | 5 | 4.7 | 4 | 8 |  |
| Borrow or receive cash |  |  |  |  |  |  |  |
| No | 291 | 67.8 | 93 | 87.7 | 41 | 82 | <0.001 |
| Yes | 138 | 32.2 | 13 | 12.3 | 9 | 18 |  |
| Sell assets |  |  |  |  |  |  |  |
| No | 403 | 93.9 | 105 | 99.1 | 47 | 94 | 0.097 |
| Yes | 26 | 6.1 | 1 | 0.9 | 3 | 6 |  |

Notes: IQR=Interquartile Range; TB=Tuberculosis; ¥ Chi-squared test; ⱡ Includes treatment failure, loss to follow-up, transfer out and death.

**Table S2a. EQ-5D-5L dimensions, utility index and EQ-VAS score by care pathway, intensive phase.**

| **Intensive phase** | **NTP (N=429)** | | **ACF (N=106)** | | **PPM (N=50)** | |  |
| --- | --- | --- | --- | --- | --- | --- | --- |
|  | N | % | N | % | N | % | p-value^¥^ |
| **MOBILITY** |  |  |  |  |  |  |  |
| No problems | 284 | 66.2 | 83 | 80.0 | 40 | 69.6 | 0.196 |
| Any problems | 145 | 33.8 | 23 | 20.0 | 10 | 30.4 |  |
| Slight problems | 108 | 25.2 | 16 | 16.0 | 8 | 22.6 |  |
| Moderate problems | 13 | 3.0 | 1 | 2.0 | 1 | 2.6 |  |
| Severe problems | 21 | 4.9 | 6 | 2.0 | 1 | 4.8 |  |
| Unable to walk about | 3 | 0.7 | 0 | 0.0 | 0 | 0.5 |  |
| **SELF-CARE** |  |  |  |  |  |  |  |
| No problems | 389 | 90.7 | 96 | 88.0 | 44 | 90.4 | 0.975 |
| Any problems | 40 | 9.3 | 10 | 12.0 | 6 | 9.6 |  |
| Slight problems | 29 | 6.8 | 7 | 10.0 | 5 | 7.0 |  |
| Moderate problems | 4 | 0.9 | 1 | 0.0 | 0 | 0.9 |  |
| Severe problems | 5 | 1.2 | 1 | 2.0 | 1 | 1.2 |  |
| Unable to wash or dress myself | 2 | 0.5 | 1 | 0.0 | 0 | 0.5 |  |
| **USUAL ACTIVITIES** |  |  |  |  |  |  |  |
| No problems | 275 | 64.1 | 78 | 68.0 | 34 | 66.2 | 0.521 |
| Any problems | 154 | 35.9 | 28 | 32.0 | 16 | 33.8 |  |
| Slight problems | 95 | 22.1 | 21 | 24.0 | 12 | 21.9 |  |
| Moderate problems | 19 | 4.4 | 2 | 2.0 | 1 | 3.8 |  |
| Severe problems | 21 | 4.9 | 4 | 2.0 | 1 | 4.4 |  |
| Unable to do | 19 | 4.4 | 1 | 4.0 | 2 | 3.8 |  |
| **PAIN/DISCOMFORT** |  |  |  |  |  |  |  |
| No pain | 184 | 42.9 | 59 | 54.0 | 27 | 46.2 | 0.257 |
| Any pain | 245 | 57.1 | 47 | 46.0 | 23 | 53.8 |  |
| Slight pain | 173 | 40.3 | 34 | 40.0 | 20 | 38.8 |  |
| Moderate pain | 35 | 8.2 | 7 | 2.0 | 1 | 7.4 |  |
| Severe pain | 34 | 7.9 | 6 | 4.0 | 2 | 7.2 |  |
| Extreme pain | 3 | 0.7 | 0 | 0.0 | 0 | 0.5 |  |
| **ANXIETY/DEPRESSION** |  |  |  |  |  |  |  |
| Not anxious or depressed | 266 | 62.0 | 79 | 70.0 | 35 | 65.0 | 0.186 |
| Any anxiety or depression | 163 | 38.0 | 27 | 30.0 | 15 | 35.0 |  |
| Slightly | 101 | 23.5 | 15 | 22.0 | 11 | 21.7 |  |
| Moderately | 25 | 5.8 | 5 | 8.0 | 4 | 5.8 |  |
| Severely | 35 | 8.2 | 7 | 0.0 | 0 | 7.2 |  |
| Extremely | 2 | 0.5 | 0 | 0.0 | 0 | 0.3 |  |
| **COMPOSITE INDICATORS** |  |  |  |  |  |  |  |
| Utility index (mean [95%CI]) | 0.82 | [0.80, 0.84] | 0.87 | [0.84, 0.91] | 0.88 | [0.83, 0.92] | |
| EQ-VAS score (mean [95%CI]) | 65.5 | [63.7, 67.3] | 70.4 | [67.1, 73.7] | 73.9 | [69.2, 78.7] | |

Notes: CI=confidence interval; ¥ Chi-squared test.

**Table S2b. EQ-5D-5L dimensions, utility index and EQ-VAS score by care pathway, continuation phase.**

| **Continuation phase** | **NTP (N=429)** | | **ACF (N=106)** | | **PPM (N=50)** | |  |
| --- | --- | --- | --- | --- | --- | --- | --- |
|  | N | % | N | % | N | % | p-value^¥^ |
| **MOBILITY** |  |  |  |  |  |  |  |
| No problems | 321 | 74.8 | 79 | 84.0 | 42 | 75.6 | 0.295 |
| Any problems | 108 | 25.2 | 27 | 16.0 | 8 | 24.5 |  |
| Slight problems | 82 | 19.1 | 17 | 8.0 | 4 | 17.6 |  |
| Moderate problems | 10 | 2.3 | 7 | 4.0 | 2 | 3.3 |  |
| Severe problems | 14 | 3.3 | 3 | 4.0 | 2 | 3.3 |  |
| Unable to walk about | 2 | 0.5 | 0 | 0.0 | 0 | 0.3 |  |
| **SELF-CARE** |  |  |  |  |  |  |  |
| No problems | 389 | 90.7 | 93 | 90.0 | 45 | 90.1 | 0.649 |
| Any problems | 40 | 9.3 | 13 | 10.0 | 5 | 9.9 |  |
| Slight problems | 30 | 7.0 | 9 | 8.0 | 4 | 7.4 |  |
| Moderate problems | 5 | 1.2 | 1 | 2.0 | 1 | 1.2 |  |
| Severe problems | 1 | 0.2 | 2 | 0.0 | 0 | 0.5 |  |
| Unable to wash or dress myself | 4 | 0.9 | 1 | 0.0 | 0 | 0.9 |  |
| **USUAL ACTIVITIES** |  |  |  |  |  |  |  |
| No problems | 327 | 76.2 | 85 | 74.0 | 37 | 76.8 | 0.176 |
| Any problems | 102 | 23.8 | 21 | 26.0 | 13 | 23.2 |  |
| Slight problems | 77 | 18.0 | 13 | 16.0 | 8 | 16.8 |  |
| Moderate problems | 5 | 1.2 | 5 | 6.0 | 3 | 2.2 |  |
| Severe problems | 12 | 2.8 | 1 | 2.0 | 1 | 2.4 |  |
| Unable to do | 8 | 1.9 | 2 | 2.0 | 1 | 1.9 |  |
| **PAIN/DISCOMFORT** |  |  |  |  |  |  |  |
| No pain | 224 | 52.2 | 66 | 68.0 | 34 | 55.4 | 0.223 |
| Any pain | 205 | 47.8 | 40 | 32.0 | 16 | 44.6 |  |
| Slight pain | 139 | 32.4 | 24 | 24.0 | 12 | 29.9 |  |
| Moderate pain | 35 | 8.2 | 9 | 2.0 | 1 | 7.7 |  |
| Severe pain | 26 | 6.1 | 7 | 6.0 | 3 | 6.2 |  |
| Extreme pain | 5 | 1.2 | 0 | 0.0 | 0 | 0.9 |  |
| **ANXIETY/DEPRESSION** |  |  |  |  |  |  |  |
| Not anxious or depressed | 297 | 69.2 | 78 | 78.0 | 39 | 70.8 | 0.810 |
| Any anxiety or depression | 132 | 30.8 | 28 | 22.0 | 11 | 29.2 |  |
| Slightly | 81 | 18.9 | 17 | 16.0 | 8 | 18.1 |  |
| Moderately | 26 | 6.1 | 5 | 4.0 | 2 | 5.6 |  |
| Severely | 20 | 4.7 | 6 | 2.0 | 1 | 4.6 |  |
| Extremely | 5 | 1.2 | 0 | 0.0 | 0 | 0.9 |  |
| **COMPOSITE INDICATORS** |  |  |  |  |  |  |  |
| Utility index (mean [95%CI]) | 0.86 | [0.84, 0.88] | 0.88 | [0.84, 0.91] | 0.90 | [0.86, 0.94] | |
| EQ-VAS score (mean [95%CI]) | 70.5 | [68.9, 72.1] | 69.6 | [66.5, 72.6] | 76.3 | [71.8, 80.7] | |

Notes: CI=confidence interval; ¥ Chi-squared test.

**Table S2c. EQ-5D-5L dimensions, utility index and EQ-VAS score by care pathway, end of treatment.**

| **End of treatment** | **NTP (N=429)** | | **ACF (N=106)** | | **PPM (N=50)** | |  |
| --- | --- | --- | --- | --- | --- | --- | --- |
|  | N | % | N | % | N | % | p-value^¥^ |
| **MOBILITY** |  |  |  |  |  |  |  |
| No problems | 363 | 84.6 | 87 | 88.0 | 44 | 84.4 | 0.673 |
| Any problems | 66 | 15.4 | 19 | 12.0 | 6 | 15.6 |  |
| Slight problems | 38 | 8.9 | 11 | 10.0 | 5 | 9.2 |  |
| Moderate problems | 12 | 2.8 | 4 | 0.0 | 0 | 2.7 |  |
| Severe problems | 14 | 3.3 | 3 | 2.0 | 1 | 3.1 |  |
| Unable to walk about | 2 | 0.5 | 1 | 0.0 | 0 | 0.5 |  |
| **SELF-CARE** |  |  |  |  |  |  |  |
| No problems | 403 | 93.9 | 91 | 94.0 | 47 | 92.5 | 0.146 |
| Any problems | 26 | 6.1 | 15 | 6.0 | 3 | 7.5 |  |
| Slight problems | 14 | 3.3 | 10 | 6.0 | 3 | 4.6 |  |
| Moderate problems | 5 | 1.2 | 1 | 0.0 | 0 | 1.0 |  |
| Severe problems | 4 | 0.9 | 3 | 0.0 | 0 | 1.2 |  |
| Unable to wash or dress myself | 3 | 0.7 | 1 | 0.0 | 0 | 0.7 |  |
| **USUAL ACTIVITIES** |  |  |  |  |  |  |  |
| No problems | 376 | 87.7 | 89 | 90.0 | 45 | 87.2 | 0.727 |
| Any problems | 53 | 12.4 | 17 | 10.0 | 5 | 12.8 |  |
| Slight problems | 26 | 6.1 | 10 | 8.0 | 4 | 6.8 |  |
| Moderate problems | 10 | 2.3 | 1 | 2.0 | 1 | 2.1 |  |
| Severe problems | 11 | 2.6 | 4 | 0.0 | 0 | 2.6 |  |
| Unable to do | 6 | 1.4 | 2 | 0.0 | 0 | 1.4 |  |
| **PAIN/DISCOMFORT** |  |  |  |  |  |  |  |
| No pain | 303 | 70.6 | 71 | 78.0 | 39 | 70.6 | 0.867 |
| Any pain | 126 | 29.4 | 35 | 22.0 | 11 | 29.4 |  |
| Slight pain | 87 | 20.3 | 23 | 14.0 | 7 | 20.0 |  |
| Moderate pain | 16 | 3.7 | 4 | 2.0 | 1 | 3.6 |  |
| Severe pain | 21 | 4.9 | 8 | 6.0 | 3 | 5.5 |  |
| Extreme pain | 2 | 0.5 | 0 | 0.0 | 0 | 0.3 |  |
| **ANXIETY/DEPRESSION** |  |  |  |  |  |  |  |
| Not anxious or depressed | 330 | 76.9 | 78 | 86.0 | 43 | 77.1 | 0.345 |
| Any anxiety or depression | 99 | 23.1 | 28 | 14.0 | 7 | 22.9 |  |
| Slightly | 56 | 13.1 | 20 | 8.0 | 4 | 13.7 |  |
| Moderately | 20 | 4.7 | 3 | 6.0 | 3 | 4.4 |  |
| Severely | 19 | 4.4 | 3 | 0.0 | 0 | 3.8 |  |
| Extremely | 4 | 0.9 | 2 | 0.0 | 0 | 1.0 |  |
| **COMPOSITE INDICATORS** |  |  |  |  |  |  |  |
| Utility index (mean [95%CI]) | 0.90 | [0.89, 0.92] | 0.88 | [0.84, 0.93] | 0.94 | [0.91, 0.97] | |
| EQ-VAS score (mean [95%CI]) | 79.5 | [78.0, 80.9] | 77.7 | [74.8, 80.7] | 82.0 | [78.1, 85.9] | |

Notes: CI=confidence interval; ¥ Chi-squared test.

**Table S3. Post-hoc pairwise comparison of mean differences in utility indexes and EQ-VAS scores.**

| Mean difference *p-value* | **Utility indexes** | | | | **EQ-VAS** | | | |
| --- | --- | --- | --- | --- | --- | --- | --- | --- |
| *Age* |  |  |  |  |  |  |  |  |
| **Intensive phase** | **< 35 years** | **35-44 years** | **45-54 years** | **55-64 years** | **< 35 years** | **35-44 years** | **45-54 years** | **55-64 years** |
| **35-44 years** | 1.96 |  |  |  | 3.42 |  |  |  |
|  | *0.247* |  |  |  | *0.001* |  |  |  |
| **45-54 years** | 1.15 | -0.84 |  |  | 2.71 | -0.79 |  |  |
|  | *0.419* | *0.403* |  |  | *0.009* | *0.238* |  |  |
| **55-64 years** | 1.38 | -0.76 | 0.14 |  | 3.85 | 0.01 | 0.89 |  |
|  | *0.415* | *0.319* | *0.444* |  | *0.000* | *0.498* | *0.233* |  |
| **65+ years** | 0.79 | -1.06 | -0.28 | -0.43 | 4.54 | 1.13 | 1.94 | 1.24 |
|  | *0.359* | *0.361* | *0.433* | *0.418* | *<0.001* | *0.185* | *0.052* | *0.180* |
| **Continuation phase** | **< 35 years** | **35-44 years** | **45-54 years** | **55-64 years** | **< 35 years** | **35-44 years** | **45-54 years** | **55-64 years** |
| **35-44 years** | 3.35 |  |  |  | 3.46 |  |  |  |
|  | *0.001* |  |  |  | *0.001* |  |  |  |
| **45-54 years** | 3.53 | 0.05 |  |  | 5.30 | 1.59 |  |  |
|  | *0.001* | *0.481* |  |  | *<0.001* | *0.070* |  |  |
| **55-64 years** | 5.10 | 1.23 | 1.23 |  | 6.66 | 2.55 | 0.89 |  |
|  | *<0.001* | *0.217* | *0.181* |  | *<0.001* | *0.009* | *0.186* |  |
| **65+ years** | 3.72 | 0.43 | 0.40 | -0.73 | 6.79 | 3.21 | 1.76 | 1.05 |
|  | *0.001* | *0.417* | *0.385* | *0.333* | *<0.001* | *0.001* | *0.056* | *0.164* |
| **End of treatment** | **< 35 years** | **35-44 years** | **45-54 years** | **55-64 years** | **< 35 years** | **35-44 years** | **45-54 years** | **55-64 years** |
| **35-44 years** | 1.66 |  |  |  | 4.82 |  |  |  |
|  | *0.069* |  |  |  | *<0.001* |  |  |  |
| **45-54 years** | 3.78 | 1.91 |  |  | 4.63 | -0.35 |  |  |
|  | *0.000* | *0.046* |  |  | *<0.001* | *0.363* |  |  |
| **55-64 years** | 5.31 | 3.18 | 1.18 |  | 7.24 | 1.68 | 2.15 |  |
|  | *<0.001* | *0.002* | *0.133* |  | *<0.001* | *0.058* | *0.023* |  |
| **65+ years** | 5.13 | 3.30 | 1.54 | 0.54 | 7.01 | 2.17 | 2.59 | 0.75 |
|  | *<0.001* | *0.001* | *0.077* | *0.294* | *<0.001* | *0.025* | *0.010* | *0.251* |
| *City* |  |  |  |  |  |  |  |  |
| **Intensive phase** | **HN** | **HP** | **HCMC** |  | **HN** | **HP** | **HCMC** |  |
| **HP** | -1.48 |  |  |  | 0.85 |  |  |  |
|  | *0.083* |  |  |  | *0.237* |  |  |  |
| **HCMC** | 0.05 | 2.17 |  |  | 2.50 | 1.66 |  |  |
|  | *0.479* | *0.030* |  |  | *0.038* | *0.097* |  |  |
| **DN** | -2.51 | -1.57 | -2.90 |  | -0.56 | -1.18 | -2.20 |  |
|  | *0.018* | *0.088* | *0.011* |  | *0.289* | *0.179* | *0.042* |  |
| **Continuation phase** | **HN** | **HP** | **HCMC** |  | **HN** | **HP** | **HCMC** |  |
| **HP** | -2.56 |  |  |  | 0.99 |  |  |  |
|  | *0.016* |  |  |  | *0.194* |  |  |  |
| **HCMC** | -2.47 | 0.80 |  |  | 0.34 | -1.02 |  |  |
|  | *0.013* | *0.212* |  |  | *0.369* | *0.230* |  |  |
| **DN** | -2.69 | -0.99 | -1.53 |  | -1.80 | -2.57 | -2.27 |  |
|  | *0.021* | *0.192* | *0.095* |  | *0.071* | *0.031* | *0.035* |  |
| **End of treatment** | **HN** | **HP** | **HCMC** |  | **HN** | **HP** | **HCMC** |  |
| **HP** | -1.71 |  |  |  | -0.07 |  |  |  |
|  | *0.130* |  |  |  | *0.471* |  |  |  |
| **HCMC** | -1.83 | 0.34 |  |  | -1.48 | -1.60 |  |  |
|  | *0.204* | *0.368* |  |  | *0.104* | *0.110* |  |  |
| **DN** | -1.61 | -0.47 | -0.70 |  | -2.01 | -2.04 | -1.37 |  |
|  | *0.106* | *0.383* | *0.363* |  | *0.066* | *0.124* | *0.102* |  |
| *Patient pathway* |  |  |  |  |  |  |  |  |
| **Intensive phase** | **NTP** | **ACF** |  |  | **NTP** | **ACF** |  |  |
| **ACF** | -3.14 |  |  |  | -2.32 |  |  |  |
|  | *0.003* |  |  |  | *0.015* |  |  |  |
| **PPM** | -1.68 | 0.52 |  |  | -3.08 | -1.21 |  |  |
|  | *0.070* | *0.300* |  |  | *0.003* | *0.113* |  |  |
| **Continuation phase** | **NTP** | **ACF** |  |  | **NTP** | **ACF** |  |  |
| **ACF** | -1.04 |  |  |  | 0.67 |  |  |  |
|  | *0.223* |  |  |  | *0.253* |  |  |  |
| **PPM** | -1.66 | -0.79 |  |  | -2.53 | -2.63 |  |  |
|  | *0.145* | *0.215* |  |  | *0.008* | *0.013* |  |  |
| **End of treatment** | **NTP** | **ACF** |  |  | **NTP** | **ACF** |  |  |
| **ACF** | 0.64 |  |  |  | 1.11 |  |  |  |
|  | *0.261* |  |  |  | *0.134* |  |  |  |
| **PPM** | -1.34 | -1.57 |  |  | -1.16 | -1.71 |  |  |
|  | *0.136* | *0.175* |  |  | *0.185* | *0.131* |  |  |
| *Diagnostic delay* |  |  |  |  |  |  |  |  |
| **Intensive phase** | **1-3 weeks** | **4-5 weeks** | **6-10 weeks** | **11-21 weeks** | **1-3 weeks** | **4-5 weeks** | **6-10 weeks** | **11-21 weeks** |
| **4-5 weeks** | 1.56 |  |  |  | -0.23 |  |  |  |
|  | *0.084* |  |  |  | *0.409* |  |  |  |
| **6-10 weeks** | 2.53 | 0.83 |  |  | 1.21 | 1.44 |  |  |
|  | *0.014* | *0.226* |  |  | *0.141* | *0.107* |  |  |
| **11-21 weeks** | 4.59 | 2.89 | 2.26 |  | 2.90 | 3.10 | 1.84 |  |
|  | *<0.001* | *0.010* | *0.024* |  | *0.009* | *0.010* | *0.066* |  |
| **22+ weeks** | 2.80 | 1.14 | 0.36 | -1.83 | 2.64 | 2.84 | 1.57 | -0.23 |
|  | *0.009* | *0.160* | *0.360* | *0.056* | *0.010* | *0.007* | *0.097* | *0.454* |
| **Continuation phase** | **1-3 weeks** | **4-5 weeks** | **6-10 weeks** | **11-21 weeks** | **1-3 weeks** | **4-5 weeks** | **6-10 weeks** | **11-21 weeks** |
| **4-5 weeks** | 1.36 |  |  |  | 0.51 |  |  |  |
|  | *0.145* |  |  |  | *0.338* |  |  |  |
| **6-10 weeks** | 2.07 | 0.59 |  |  | 1.22 | 0.65 |  |  |
|  | *0.064* | *0.308* |  |  | *0.160* | *0.321* |  |  |
| **11-21 weeks** | 3.15 | 1.67 | 1.19 |  | 2.66 | 2.08 | 1.57 |  |
|  | *0.008* | *0.118* | *0.167* |  | *0.020* | *0.046* | *0.097* |  |
| **22+ weeks** | 2.92 | 1.47 | 0.98 | -0.19 | 2.71 | 2.14 | 1.64 | 0.09 |
|  | *0.009* | *0.141* | *0.206* | *0.424* | *0.034* | *0.054* | *0.101* | *0.463* |
| **End of treatment** | **1-3 weeks** | **4-5 weeks** | **6-10 weeks** | **11-21 weeks** | **1-3 weeks** | **4-5 weeks** | **6-10 weeks** | **11-21 weeks** |
| **4-5 weeks** | 0.50 |  |  |  | 1.43 |  |  |  |
|  | *0.342* |  |  |  | *0.127* |  |  |  |
| **6-10 weeks** | 0.67 | 0.12 |  |  | 1.03 | -0.51 |  |  |
|  | *0.361* | *0.451* |  |  | *0.188* | *0.306* |  |  |
| **11-21 weeks** | 3.18 | 2.61 | 2.72 |  | 2.59 | 1.05 | 1.69 |  |
|  | *0.007* | *0.015* | *0.016* |  | *0.024* | *0.210* | *0.113* |  |
| **22+ weeks** | 2.58 | 2.03 | 2.08 | -0.59 | 3.21 | 1.68 | 2.37 | 0.69 |
|  | *0.012* | *0.036* | *0.038* | *0.348* | *0.007* | *0.093* | *0.030* | *0.272* |
| *Health-seeking* |  |  |  |  |  |  |  |  |
| **Intensive phase** | **1-2 attempts** | **3-4 attempts** | **5-6 attempts** | **7-10 attempts** | **1-2 attempts** | **3-4 attempts** | **5-6 attempts** | **7-10 attempts** |
| **3-4 attempts** | 0.84 |  |  |  | -0.16 |  |  |  |
|  | *0.250* |  |  |  | *0.437* |  |  |  |
| **5-6 attempts** | 1.49 | 0.81 |  |  | 0.27 | 0.50 |  |  |
|  | *0.114* | *0.233* |  |  | *0.438* | *0.388* |  |  |
| **7-10 attempts** | 1.55 | 0.88 | 0.06 |  | 0.92 | 1.27 | 0.72 |  |
|  | *0.121* | *0.272* | *0.478* |  | *0.298* | *0.204* | *0.335* |  |
| **11+ attempts** | 4.09 | 3.89 | 2.88 | 2.85 | 2.51 | 3.16 | 2.49 | 1.79 |
|  | *0.000* | *0.000* | *0.007* | *0.005* | *0.031* | *0.008* | *0.021* | *0.093* |
| **Continuation phase** | **1-2 attempts** | **3-4 attempts** | **5-6 attempts** | **7-10 attempts** | **1-2 attempts** | **3-4 attempts** | **5-6 attempts** | **7-10 attempts** |
| **3-4 attempts** | -1.15 |  |  |  | -0.96 |  |  |  |
|  | *0.248* |  |  |  | *0.337* |  |  |  |
| **5-6 attempts** | -1.31 | -0.25 |  |  | -0.65 | 0.32 |  |  |
|  | *0.238* | *0.447* |  |  | *0.371* | *0.375* |  |  |
| **7-10 attempts** | -1.02 | 0.10 | 0.33 |  | 0.32 | 1.48 | 1.08 |  |
|  | *0.219* | *0.459* | *0.462* |  | *0.415* | *0.233* | *0.348* |  |
| **11+ attempts** | 1.06 | 2.58 | 2.66 | 2.35 | 0.89 | 2.16 | 1.73 | 0.64 |
|  | *0.241* | *0.024* | *0.039* | *0.031* | *0.310* | *0.153* | *0.211* | *0.325* |
| **End of treatment** | **1-2 attempts** | **3-4 attempts** | **5-6 attempts** | **7-10 attempts** | **1-2 attempts** | **3-4 attempts** | **5-6 attempts** | **7-10 attempts** |
| **3-4 attempts** | -1.05 |  |  |  | -0.10 |  |  |  |
|  | *0.293* |  |  |  | *0.658* |  |  |  |
| **5-6 attempts** | -0.80 | 0.23 |  |  | 0.16 | 0.29 |  |  |
|  | *0.352* | *0.407* |  |  | *1.000* | *1.000* |  |  |
| **7-10 attempts** | -0.54 | 0.56 | 0.30 |  | 0.14 | 0.28 | -0.02 |  |
|  | *0.368* | *0.413* | *0.425* |  | *0.740* | *1.000* | *0.617* |  |
| **11+ attempts** | 1.26 | 2.70 | 2.31 | 2.03 | 0.15 | 0.29 | -0.01 | 0.01 |
|  | *0.261* | *0.035* | *0.052* | *0.070* | *0.881* | *1.000* | *0.552* | *0.497* |
| *Education level* |  |  |  |  |  |  |  |  |
| **Intensive phase** | **Not literate** | **Primary school** | **Secondary school** | **High school** | **Not literate** | **Primary school** | **Secondary school** | **High school** |
| **Primary school** | -2.37 |  |  |  | -2.58 |  |  |  |
|  | *0.018* |  |  |  | *0.008* |  |  |  |
| **Secondary school** | -3.52 | -2.45 |  |  | -3.78 | -2.54 |  |  |
|  | *0.002* | *0.018* |  |  | *0.000* | *0.008* |  |  |
| **High school** | -2.71 | -0.85 | 1.27 |  | -4.57 | -3.97 | -1.59 |  |
|  | *0.011* | *0.247* | *0.145* |  | *0.000* | *0.000* | *0.062* |  |
| **University/Post-graduate** | -3.10 | -1.60 | 0.35 | -0.75 | -4.73 | -4.11 | -1.97 | -0.50 |
|  | *0.005* | *0.091* | *0.364* | *0.251* | *0.000* | *0.000* | *0.030* | *0.308* |
| **Continuation phase** | **Not literate** | **Primary school** | **Secondary school** | **High school** | **Not literate** | **Primary school** | **Secondary school** | **High school** |
| **Primary school** | -2.09 |  |  |  | -0.27 |  |  |  |
|  | *0.092* |  |  |  | *0.393* |  |  |  |
| **Secondary school** | -1.65 | 0.82 |  |  | -0.92 | -1.33 |  |  |
|  | *0.124* | *0.295* |  |  | *0.199* | *0.114* |  |  |
| **High school** | -1.98 | 0.02 | -0.68 |  | -1.97 | -3.24 | -1.92 |  |
|  | *0.079* | *0.492* | *0.277* |  | *0.049* | *0.002* | *0.039* |  |
| **University/Post-graduate** | -2.41 | -0.84 | -1.43 | -0.77 | -3.16 | -5.08 | -3.82 | -1.96 |
|  | *0.080* | *0.333* | *0.152* | *0.276* | *0.002* | *0.000* | *0.000* | *0.042* |
| **End of treatment** | **Not literate** | **Primary school** | **Secondary school** | **High school** | **Not literate** | **Primary school** | **Secondary school** | **High school** |
| **Primary school** | -1.76 |  |  |  | -1.03 |  |  |  |
|  | *0.056* |  |  |  | *0.168* |  |  |  |
| **Secondary school** | -2.00 | -0.55 |  |  | -1.21 | -0.41 |  |  |
|  | *0.038* | *0.323* |  |  | *0.141* | *0.340* |  |  |
| **High school** | -2.02 | -0.63 | -0.13 |  | -2.18 | -2.27 | -1.79 |  |
|  | *0.044* | *0.329* | *0.449* |  | *0.024* | *0.023* | *0.053* |  |
| **University/Post-graduate** | -3.46 | -3.21 | -2.64 | -2.36 | -3.65 | -4.71 | -4.18 | -2.40 |
|  | *0.003* | *0.003* | *0.014* | *0.023* | *0.000* | *0.000* | *0.000* | *0.020* |
| *Education length* |  |  |  |  |  |  |  |  |
| **Intensive phase** | **0-4 years** | **5-6 years** | **7-8 years** | **9-11 years** | **0-4 years** | **5-6 years** | **7-8 years** | **9-11 years** |
| **5-6 years** | -0.69 |  |  |  | -2.58 |  |  |  |
|  | *0.305* |  |  |  | *0.008* |  |  |  |
| **7-8 years** | -1.76 | -1.06 |  |  | -3.78 | -2.54 |  |  |
|  | *0.130* | *0.243* |  |  | *0.000* | *0.008* |  |  |
| **9-11 years** | -2.44 | -1.66 | -0.53 |  | -4.57 | -3.97 | -1.59 |  |
|  | *0.074* | *0.121* | *0.332* |  | *0.000* | *0.000* | *0.062* |  |
| **12+ years** | -1.87 | -1.06 | 0.13 | 0.75 | -4.73 | -4.11 | -1.97 | -0.50 |
|  | *0.152* | *0.288* | *0.448* | *0.323* | *0.000* | *0.000* | *0.030* | *0.308* |
| **Continuation phase** | **0-4 years** | **5-6 years** | **7-8 years** | **9-11 years** | **0-4 years** | **5-6 years** | **7-8 years** | **9-11 years** |
| **5-6 years** | -0.21 |  |  |  | -0.27 |  |  |  |
|  | *0.463* |  |  |  | *0.393* |  |  |  |
| **7-8 years** | 0.52 | 0.71 |  |  | -0.92 | -1.33 |  |  |
|  | *0.376* | *0.396* |  |  | *0.199* | *0.114* |  |  |
| **9-11 years** | -0.82 | -0.58 | -1.32 |  | -1.97 | -3.24 | -1.92 |  |
|  | *0.514* | *0.400* | *0.463* |  | *0.049* | *0.002* | *0.039* |  |
| **12+ years** | -1.08 | -0.81 | -1.60 | -0.21 | -3.16 | -5.08 | -3.82 | -1.96 |
|  | *0.471* | *0.416* | *0.553* | *0.418* | *0.002* | *0.000* | *0.000* | *0.042* |
| **End of treatment** | **0-4 years** | **5-6 years** | **7-8 years** | **9-11 years** | **0-4 years** | **5-6 years** | **7-8 years** | **9-11 years** |
| **5-6 years** | 0.02 |  |  |  | -1.03 |  |  |  |
|  | *0.492* |  |  |  | *0.168* |  |  |  |
| **7-8 years** | 0.08 | 0.06 |  |  | -1.21 | -0.41 |  |  |
|  | *0.584* | *0.529* |  |  | *0.141* | *0.340* |  |  |
| **9-11 years** | -1.42 | -1.40 | -1.44 |  | -2.18 | -2.27 | -1.79 |  |
|  | *0.157* | *0.135* | *0.186* |  | *0.024* | *0.023* | *0.053* |  |
| **12+ years** | -2.82 | -2.76 | -2.78 | -1.36 | -3.65 | -4.71 | -4.18 | -2.40 |
|  | *0.024* | *0.010* | *0.014* | *0.124* | *0.000* | *0.000* | *0.000* | *0.020* |
| *Household size* |  |  |  |  |  |  |  |  |
| **Intensive phase** | **1 person** | **2 persons** | **3 persons** | **4 persons** | **1 person** | **2 persons** | **3 persons** | **4 persons** |
| **2 persons** | 1.34 |  |  |  | 0.38 |  |  |  |
|  | *0.904* |  |  |  | *0.701* |  |  |  |
| **3 persons** | 0.98 | -0.51 |  |  | 0.23 | -0.21 |  |  |
|  | *0.545* | *0.381* |  |  | *0.512* | *0.461* |  |  |
| **4 persons** | 0.85 | -0.74 | -0.22 |  | -0.27 | -0.88 | -0.70 |  |
|  | *0.493* | *0.385* | *0.415* |  | *0.560* | *0.949* | *0.606* |  |
| **5+ persons** | 0.45 | -1.34 | -0.83 | -0.64 | -0.33 | -0.99 | -0.81 | -0.07 |
|  | *0.361* | *0.453* | *0.405* | *0.371* | *0.619* | *1.000* | *0.700* | *0.473* |
| **Continuation phase** | **1 person** | **2 persons** | **3 persons** | **4 persons** | **1 person** | **2 persons** | **3 persons** | **4 persons** |
| **2 persons** | -0.47 |  |  |  | 0.50 |  |  |  |
|  | *0.401* |  |  |  | *0.388* |  |  |  |
| **3 persons** | -1.26 | -0.99 |  |  | -0.22 | -0.93 |  |  |
|  | *0.345* | *0.324* |  |  | *0.412* | *0.252* |  |  |
| **4 persons** | -1.21 | -0.92 | 0.12 |  | -1.02 | -1.99 | -1.08 |  |
|  | *0.281* | *0.297* | *0.454* |  | *0.257* | *0.118* | *0.279* |  |
| **5+ persons** | -1.60 | -1.43 | -0.37 | -0.53 | -1.28 | -2.40 | -1.47 | -0.34 |
|  | *0.545* | *0.379* | *0.395* | *0.427* | *0.251* | *0.083* | *0.234* | *0.407* |
| **End of treatment** | **1 person** | **2 persons** | **3 persons** | **4 persons** | **1 person** | **2 persons** | **3 persons** | **4 persons** |
| **2 persons** | 0.22 |  |  |  | 0.93 |  |  |  |
|  | *0.459* |  |  |  | *0.251* |  |  |  |
| **3 persons** | -1.15 | -1.74 |  |  | -1.13 | -2.65 |  |  |
|  | *0.313* | *0.412* |  |  | *0.215* | *0.041* |  |  |
| **4 persons** | -1.08 | -1.69 | 0.14 |  | -0.89 | -2.41 | 0.38 |  |
|  | *0.281* | *0.229* | *0.444* |  | *0.235* | *0.039* | *0.391* |  |
| **5+ persons** | -0.67 | -1.20 | 0.78 | 0.67 | -0.13 | -1.52 | 1.51 | 1.18 |
|  | *0.315* | *0.385* | *0.363* | *0.357* | *0.447* | *0.213* | *0.163* | *0.237* |
| *Employment* | | | | | | | | |
| **Intensive phase** | **Unemployed** | **Formally employed** | **Informally employed** |  | **Unemployed** | **Formally employed** | **Informally employed** |  |
| **Formally employed** | -0.86 |  |  |  | -2.83 |  |  |  |
|  | *0.588* |  |  |  | *0.007* |  |  |  |
| **Informally employed** | -0.25 | 0.72 |  |  | -0.24 | 2.83 |  |  |
|  | *0.402* | *0.470* |  |  | *0.405* | *0.005* |  |  |
| **Don't know/No answer** | 0.38 | 1.05 | 0.60 |  | 0.51 | 2.83 | 0.72 |  |
|  | *0.420* | *0.875* | *0.414* |  | *0.367* | *0.014* | *0.353* |  |
| **Continuation phase** | **Unemployed** | **Formally employed** | **Informally employed** |  | **Unemployed** | **Formally employed** | **Informally employed** |  |
| **Formally employed** | -1.48 |  |  |  | -3.97 |  |  |  |
|  | *0.208* |  |  |  | *0.000* |  |  |  |
| **Informally employed** | -0.36 | 1.30 |  |  | -2.80 | 2.10 |  |  |
|  | *0.359* | *0.193* |  |  | *0.004* | *0.022* |  |  |
| **Don't know/No answer** | 0.96 | 2.08 | 1.29 |  | 1.03 | 4.24 | 3.20 |  |
|  | *0.203* | *0.114* | *0.148* |  | *0.152* | *0.000* | *0.001* |  |
| **End of treatment** | **Unemployed** | **Formally employed** | **Informally employed** |  | **Unemployed** | **Formally employed** | **Informally employed** |  |
| **Formally employed** | -3.54 |  |  |  | -3.98 |  |  |  |
|  | *0.001* |  |  |  | *0.000* |  |  |  |
| **Informally employed** | -2.50 | 1.87 |  |  | -2.09 | 2.65 |  |  |
|  | *0.012* | *0.037* |  |  | *0.022* | *0.006* |  |  |
| **Don't know/No answer** | 0.57 | 3.49 | 2.49 |  | 1.15 | 4.36 | 2.79 |  |
|  | *0.283* | *0.001* | *0.010* |  | *0.126* | *0.000* | *0.005* |  |
| *Pre-TB monthly income* | | | | | | | | |
| **Intensive phase** | **USD 0-24** | **USD 25-169** | **USD 170-259** | **USD 260-389** | **USD 0-24** | **USD 25-169** | **USD 170-259** | **USD 260-389** |
| **USD 25-169** | -0.34 |  |  |  | -1.66 |  |  |  |
|  | *0.523* |  |  |  | *0.122* |  |  |  |
| **USD 170-259** | -0.37 | -0.02 |  |  | -1.05 | 0.64 |  |  |
|  | *0.595* | *0.492* |  |  | *0.247* | *0.291* |  |  |
| **USD 260-389** | -0.41 | -0.07 | -0.05 |  | -2.00 | -0.35 | -0.99 |  |
|  | *0.682* | *0.591* | *0.533* |  | *0.115* | *0.364* | *0.231* |  |
| **USD 390+** | -0.98 | -0.64 | -0.63 | -0.57 | -2.75 | -1.12 | -1.76 | -0.77 |
|  | *1.000* | *1.000* | *0.878* | *0.710* | *0.030* | *0.264* | *0.130* | *0.276* |
| **Continuation phase** | **USD 0-24** | **USD 25-169** | **USD 170-259** | **USD 260-389** | **USD 0-24** | **USD 25-169** | **USD 170-259** | **USD 260-389** |
| **USD 25-169** | 0.27 |  |  |  | -1.79 |  |  |  |
|  | *0.493* |  |  |  | *0.062* |  |  |  |
| **USD 170-259** | 0.49 | 0.22 |  |  | -1.88 | -0.06 |  |  |
|  | *0.444* | *0.412* |  |  | *0.075* | *0.475* |  |  |
| **USD 260-389** | -1.63 | -1.89 | -2.14 |  | -3.69 | -1.91 | -1.88 |  |
|  | *0.173* | *0.146* | *0.160* |  | *0.001* | *0.094* | *0.061* |  |
| **USD 390+** | -0.24 | -0.51 | -0.73 | 1.36 | -3.18 | -1.42 | -1.38 | 0.47 |
|  | *0.450* | *0.511* | *0.464* | *0.215* | *0.004* | *0.112* | *0.105* | *0.354* |
| **End of treatment** | **USD 0-24** | **USD 25-169** | **USD 170-259** | **USD 260-389** | **USD 0-24** | **USD 25-169** | **USD 170-259** | **USD 260-389** |
| **USD 25-169** | -0.45 |  |  |  | -1.19 |  |  |  |
|  | *0.327* |  |  |  | *0.196* |  |  |  |
| **USD 170-259** | -1.13 | -0.67 |  |  | -2.09 | -0.89 |  |  |
|  | *0.186* | *0.279* |  |  | *0.061* | *0.268* |  |  |
| **USD 260-389** | -3.36 | -2.91 | -2.29 |  | -2.49 | -1.31 | -0.45 |  |
|  | *0.004* | *0.009* | *0.037* |  | *0.032* | *0.191* | *0.364* |  |
| **USD 390+** | -2.04 | -1.60 | -0.97 | 1.28 | -2.63 | -1.46 | -0.61 | -0.16 |
|  | *0.051* | *0.110* | *0.209* | *0.167* | *0.043* | *0.181* | *0.340* | *0.436* |
| *Decline in monthly income* | | | | | | | | |
| **Intensive phase** | **No decline** | **USD 1-100** | **USD 101-250** | **USD 251-400** | **No decline** | **USD 1-100** | **USD 101-250** | **USD 251-400** |
| **USD 1-100** | 2.72 |  |  |  | 1.00 |  |  |  |
|  | *0.016* |  |  |  | *0.797* |  |  |  |
| **USD 101-250** | 1.89 | -0.68 |  |  | 0.47 | -0.43 |  |  |
|  | *0.073* | *0.309* |  |  | *0.530* | *0.475* |  |  |
| **USD 251-400** | 4.17 | 1.22 | 1.90 |  | 1.64 | 0.54 | 0.97 |  |
|  | *0.000* | *0.159* | *0.096* |  | *0.505* | *0.589* | *0.552* |  |
| **USD 401+** | 1.86 | -0.43 | 0.18 | -1.53 | 0.40 | -0.40 | -0.01 | -0.88 |
|  | *0.063* | *0.370* | *0.427* | *0.105* | *0.430* | *0.384* | *0.497* | *0.471* |
| **Continuation phase** | **No decline** | **USD 1-100** | **USD 101-250** | **USD 251-400** | **No decline** | **USD 1-100** | **USD 101-250** | **USD 251-400** |
| **USD 1-100** | 2.07 |  |  |  | 1.66 |  |  |  |
|  | *0.064* |  |  |  | *0.480* |  |  |  |
| **USD 101-250** | 2.68 | 0.51 |  |  | 1.38 | -0.23 |  |  |
|  | *0.037* | *0.433* |  |  | *0.418* | *0.409* |  |  |
| **USD 251-400** | 1.83 | -0.20 | -0.71 |  | 0.27 | -1.16 | -0.93 |  |
|  | *0.084* | *0.469* | *0.478* |  | *0.438* | *0.410* | *0.443* |  |
| **USD 401+** | 2.24 | 0.39 | -0.08 | 0.56 | 0.85 | -0.52 | -0.31 | 0.53 |
|  | *0.063* | *0.438* | *0.469* | *0.479* | *0.398* | *0.433* | *0.475* | *0.497* |
| **End of treatment** | **No decline** | **USD 1-100** | **USD 101-250** | **USD 251-400** | **No decline** | **USD 1-100** | **USD 101-250** | **USD 251-400** |
| **USD 1-100** | 1.79 |  |  |  | 1.17 |  |  |  |
|  | *0.183* |  |  |  | *0.305* |  |  |  |
| **USD 101-250** | 1.87 | 0.07 |  |  | 2.49 | 1.11 |  |  |
|  | *0.307* | *0.471* |  |  | *0.063* | *0.265* |  |  |
| **USD 251-400** | 0.21 | -1.31 | -1.38 |  | 0.45 | -0.59 | -1.70 |  |
|  | *0.463* | *0.236* | *0.278* |  | *0.407* | *0.396* | *0.222* |  |
| **USD 401+** | 0.72 | -0.72 | -0.79 | 0.46 | 0.82 | -0.17 | -1.17 | 0.37 |
|  | *0.338* | *0.392* | *0.432* | *0.402* | *0.345* | *0.434* | *0.405* | *0.396* |

**Table 4a. Utility index by participant characteristics, care pathway and stage of treatment.**

|  | **NTP (N=429)** | | | | | | **ACF (N=106)** | | | | | | **PPM (N=50)** | | | | | |
| --- | --- | --- | --- | --- | --- | --- | --- | --- | --- | --- | --- | --- | --- | --- | --- | --- | --- | --- |
|  | Intensive Phase | | Continuation Phase | | End of Treatment | | Intensive Phase | | Continuation Phase | | End of Treatment | | Intensive Phase | | Continuation Phase | | End of Treatment | |
|  | Mean | 95%CI | Mean | 95%CI | Mean | 95%CI | Mean | 95%CI | Mean | 95%CI | Mean | 95%CI | Mean | 95%CI | Mean | 95%CI | Mean | 95%CI |
| **Total** | 0.82 | [0.80, 0.84] | 0.86 | [0.84, 0.88] | 0.90 | [0.89, 0.92] | 0.87 | [0.84, 0.91] | 0.88 | [0.84, 0.91] | 0.88 | [0.84, 0.93] | 0.88 | [0.83, 0.92] | 0.90 | [0.86, 0.94] | 0.94 | [0.91, 0.97] |
| **DEMOGRAPHICS** |  |  |  |  |  |  |  |  |  |  |  |  |  |  |  |  |  |  |
| Sex |  |  |  |  |  |  |  |  |  |  |  |  |  |  |  |  |  |  |
| Female | 0.84 | [0.82, 0.86] | 0.88 | [0.86, 0.89] | 0.92 | [0.90, 0.94] | 0.90 | [0.87, 0.93] | 0.90 | [0.87, 0.94] | 0.91 | [0.88, 0.95] | 0.87 | [0.80, 0.93] | 0.88 | [0.82, 0.95] | 0.92 | [0.87, 0.97] |
| Male | 0.76 | [0.71, 0.80] | 0.82 | [0.77, 0.86] | 0.86 | [0.82, 0.91] | 0.74 | [0.61, 0.87] | 0.76 | [0.64, 0.87] | 0.76 | [0.59, 0.92] | 0.90 | [0.86, 0.94] | 0.93 | [0.89, 0.98] | 0.98 | [0.94, 1.01] |
| Age |  |  |  |  |  |  |  |  |  |  |  |  |  |  |  |  |  |  |
| <35 years | 0.87 | [0.85, 0.90] | 0.93 | [0.92, 0.95] | 0.96 | [0.95, 0.98] | 0.88 | [0.75, 1.00] | 1.00 | [1.00, 1.00] | 0.95 | [0.84, 1.06] | 0.91 | [0.86, 0.96] | 0.96 | [0.94, 0.99] | 0.98 | [0.94, 1.01] |
| 35-44 years | 0.81 | [0.76, 0.86] | 0.87 | [0.83, 0.90] | 0.93 | [0.90, 0.96] | 0.87 | [0.73, 1.01] | 0.96 | [0.87, 1.04] | 0.98 | [0.94, 1.03] | 0.81 | [0.65, 0.97] | 0.84 | [0.67, 1.01] | 0.97 | [0.91, 1.03] |
| 45-54 years | 0.83 | [0.79, 0.87] | 0.86 | [0.82, 0.90] | 0.90 | [0.85, 0.94] | 0.82 | [0.71, 0.93] | 0.83 | [0.72, 0.94] | 0.87 | [0.75, 0.99] | 0.95 | [0.88, 1.01] | 0.95 | [0.91, 1.00] | 0.93 | [0.86, 1.01] |
| 55-64 years | 0.78 | [0.73, 0.82] | 0.81 | [0.77, 0.85] | 0.87 | [0.83, 0.91] | 0.89 | [0.84, 0.95] | 0.87 | [0.81, 0.93] | 0.88 | [0.81, 0.95] | 0.91 | [0.84, 0.98] | 0.92 | [0.84, 0.99] | 0.87 | [0.72, 1.01] |
| 65+ years | 0.78 | [0.71, 0.86] | 0.81 | [0.73, 0.88] | 0.81 | [0.72, 0.90] | 0.88 | [0.81, 0.95] | 0.89 | [0.84, 0.94] | 0.88 | [0.81, 0.95] | 0.75 | [0.53, 0.98] | 0.74 | [0.53, 0.94] | 0.92 | [0.84, 1.01] |
| City |  |  |  |  |  |  |  |  |  |  |  |  |  |  |  |  |  |  |
| Hanoi | 0.80 | [0.72, 0.88] | 0.77 | [0.70, 0.85] | 0.80 | [0.72, 0.89] | 0.90 | [0.82, 0.97] | 0.88 | [0.80, 0.97] | 0.92 | [0.83, 1.01] | 0.94 | [0.94, 0.94] | 0.97 | [0.56, 1.37] | 1.00 | [1.00, 1.00] |
| Hai Phong | 0.88 | [0.82, 0.94] | 0.90 | [0.85, 0.95] | 0.94 | [0.89, 0.99] | 0.81 | [0.67, 0.95] | 0.85 | [0.77, 0.94] | 0.85 | [0.76, 0.95] | 0.88 | [0.83, 0.94] | 0.92 | [0.87, 0.97] | 0.92 | [0.85, 1.00] |
| Ho Chi Minh City | 0.81 | [0.79, 0.83] | 0.87 | [0.85, 0.89] | 0.91 | [0.89, 0.93] | 0.88 | [0.85, 0.92] | 0.88 | [0.83, 0.93] | 0.88 | [0.82, 0.94] | 0.87 | [0.80, 0.94] | 0.89 | [0.82, 0.95] | 0.94 | [0.90, 0.98] |
| Da Nang | 0.95 | [0.90, 1.00] | 0.92 | [0.84, 1.00] | 0.93 | [0.84, 1.02] | 0.92 | [0.84, 1.00] | 0.93 | [0.84, 1.02] | 0.94 | [0.87, 1.02] | . | [., .] | . | [., .] | . | [., .] |
| **CLINICAL CHARACTERISTICS** |  |  |  |  |  |  |  |  |  |  |  |  |  |  |  |  |  |  |
| Diagnosis (N=583) |  |  |  |  |  |  |  |  |  |  |  |  |  |  |  |  |  |  |
| Bacteriologically confirmed | 0.84 | [0.73, 0.95] | 0.86 | [0.78, 0.94] | 0.88 | [0.81, 0.95] | 0.937 | [0.13, 1.74] | 0.90 | [0.21, 1.60] | 0.91 | [-0.19, 2.01] | 0.94 | [0.88, 1.00] | 0.94 | [0.89, 0.99] | 0.96 | [0.92, 1.01] |
| Clinically diagnosed | 0.82 | [0.80, 0.84] | 0.86 | [0.84, 0.88] | 0.91 | [0.89, 0.92] | 0.872 | [0.83, 0.91] | 0.88 | [0.84, 0.91] | 0.88 | [0.84, 0.93] | 0.86 | [0.81, 0.92] | 0.89 | [0.84, 0.94] | 0.93 | [0.89, 0.97] |
| TB location (N=583) |  |  |  |  |  |  |  |  |  |  |  |  |  |  |  |  |  |  |
| Pulmonary TB | 0.82 | [0.80, 0.84] | 0.86 | [0.84, 0.88] | 0.91 | [0.89, 0.92] | 0.87 | [0.84, 0.91] | 0.88 | [0.84, 0.91] | 0.88 | [0.84, 0.93] | 0.88 | [0.83, 0.92] | 0.90 | [0.86, 0.94] | 0.94 | [0.91, 0.97] |
| Extrapulmonary TB | 0.77 | [0.41, 1.13] | 0.80 | [0.54, 1.05] | 0.81 | [0.65, 0.96] | . | [., .] | . | [., .] | . | [., .] | . | [., .] | . | [., .] | . | [., .] |
| Treatment category (N=583) |  |  |  |  |  |  |  |  |  |  |  |  |  |  |  |  |  |  |
| New | 0.84 | [0.82, 0.86] | 0.87 | [0.85, 0.89] | 0.91 | [0.89, 0.93] | 0.87 | [0.84, 0.91] | 0.88 | [0.84, 0.91] | 0.88 | [0.84, 0.93] | 0.88 | [0.83, 0.92] | 0.90 | [0.86, 0.95] | 0.95 | [0.92, 0.98] |
| Retreatment | 0.75 | [0.71, 0.80] | 0.84 | [0.81, 0.87] | 0.89 | [0.84, 0.93] | . | [., .] | . | [., .] | . | [., .] | 0.86 | [0.67, 1.04] | 0.87 | [0.87, 0.87] | 0.75 | [-1.57, 3.07] |
| Treatment outcome (N=531) |  |  |  |  |  |  |  |  |  |  |  |  |  |  |  |  |  |  |
| Treatment success | 0.81 | [0.79, 0.84] | 0.86 | [0.84, 0.87] | 0.90 | [0.88, 0.92] | 0.89 | [0.86, 0.92] | 0.88 | [0.84, 0.92] | 0.89 | [0.84, 0.94] | 0.88 | [0.83, 0.92] | 0.90 | [0.86, 0.94] | 0.94 | [0.91, 0.97] |
| Unfavorable outcomeⱡ | 0.82 | [0.72, 0.91] | 0.90 | [0.83, 0.96] | 0.90 | [0.82, 0.97] | 0.93 | [0.75, 1.12] | 0.98 | [0.94, 1.03] | 0.96 | [0.91, 1.01] | . | [., .] | . | [., .] | . | [., .] |
| **HEALTH ACCESS** |  |  |  |  |  |  |  |  |  |  |  |  |  |  |  |  |  |  |
| Diagnostic delay (N=515) |  |  |  |  |  |  |  |  |  |  |  |  |  |  |  |  |  |  |
| 1-3 weeks | 0.90 | [0.87, 0.93] | 0.91 | [0.87, 0.95] | 0.96 | [0.94, 0.98] | 0.95 | [0.84, 1.06] | 0.95 | [0.88, 1.02] | 0.95 | [0.87, 1.02] | 0.90 | [0.81, 0.98] | 0.88 | [0.76, 1.00] | 0.87 | [0.71, 1.03] |
| 4-5 weeks | 0.86 | [0.82, 0.91] | 0.89 | [0.85, 0.93] | 0.92 | [0.88, 0.96] | 0.80 | [0.64, 0.96] | 0.88 | [0.79, 0.96] | 0.83 | [0.62, 1.04] | 0.91 | [0.85, 0.96] | 0.89 | [0.82, 0.97] | 0.98 | [0.94, 1.02] |
| 6-10 weeks | 0.83 | [0.79, 0.87] | 0.87 | [0.84, 0.90] | 0.94 | [0.91, 0.96] | 0.91 | [0.82, 1.00] | 0.96 | [0.92, 0.99] | 0.94 | [0.89, 1.00] | 0.80 | [0.65, 0.96] | 0.84 | [0.70, 0.99] | 0.94 | [0.89, 1.00] |
| 11-21 weeks | 0.74 | [0.69, 0.80] | 0.82 | [0.78, 0.86] | 0.85 | [0.79, 0.90] | 0.80 | [0.63, 0.96] | 0.93 | [0.88, 0.98] | 0.90 | [0.79, 1.00] | 0.89 | [0.82, 0.96] | 0.94 | [0.86, 1.01] | 0.95 | [0.86, 1.03] |
| 22+ weeks | 0.79 | [0.74, 0.84] | 0.83 | [0.78, 0.88] | 0.88 | [0.83, 0.92] | 0.88 | [0.81, 0.95] | 0.86 | [0.77, 0.94] | 0.85 | [0.72, 0.98] | 0.92 | [0.85, 0.99] | 0.96 | [0.94, 0.99] | 0.96 | [0.90, 1.03] |
| Health-seeking |  |  |  |  |  |  |  |  |  |  |  |  |  |  |  |  |  |  |
| 1-2 attempts | 0.82 | [0.73, 0.90] | 0.86 | [0.80, 0.92] | 0.91 | [0.85, 0.97] | 0.92 | [0.86, 0.97] | 0.85 | [0.76, 0.94] | 0.88 | [0.79, 0.96] | 0.82 | [0.65, 0.98] | 0.79 | [0.63, 0.95] | 0.87 | [0.75, 0.98] |
| 3-4 attempts | 0.84 | [0.80, 0.88] | 0.87 | [0.84, 0.91] | 0.93 | [0.89, 0.96] | 0.87 | [0.80, 0.94] | 0.92 | [0.89, 0.94] | 0.91 | [0.86, 0.96] | 0.90 | [0.85, 0.94] | 0.93 | [0.86, 0.99] | 0.97 | [0.94, 1.00] |
| 5-6 attempts | 0.84 | [0.80, 0.88] | 0.87 | [0.83, 0.92] | 0.91 | [0.86, 0.95] | 0.82 | [0.72, 0.91] | 0.85 | [0.76, 0.95] | 0.87 | [0.73, 1.00] | 0.92 | [0.87, 0.97] | 0.95 | [0.91, 1.00] | 0.95 | [0.87, 1.04] |
| 7-10 attempts | 0.84 | [0.80, 0.87] | 0.88 | [0.84, 0.91] | 0.91 | [0.88, 0.94] | 0.76 | [0.44, 1.08] | 0.85 | [0.67, 1.04] | 0.88 | [0.63, 1.13] | 0.94 | [0.89, 0.99] | 0.95 | [0.90, 1.00] | 0.98 | [0.94, 1.01] |
| 11+ attempts | 0.77 | [0.72, 0.81] | 0.83 | [0.79, 0.86] | 0.88 | [0.84, 0.92] | 0.86 | [0.77, 0.94] | 0.86 | [0.69, 1.03] | 0.81 | [0.53, 1.08] | 0.71 | [0.35, 1.07] | 0.85 | [0.53, 1.18] | 0.88 | [0.67, 1.10] |
| Social Health Insurance |  |  |  |  |  |  |  |  |  |  |  |  |  |  |  |  |  |  |
| No | 0.82 | [0.78, 0.86] | 0.85 | [0.81, 0.90] | 0.92 | [0.89, 0.95] | 0.83 | [0.71, 0.95] | 0.90 | [0.85, 0.95] | 0.89 | [0.79, 0.99] | 0.88 | [0.81, 0.96] | 0.96 | [0.93, 1.00] | 0.96 | [0.89, 1.03] |
| Yes | 0.82 | [0.80, 0.84] | 0.86 | [0.85, 0.88] | 0.90 | [0.88, 0.92] | 0.88 | [0.84, 0.92] | 0.87 | [0.83, 0.92] | 0.88 | [0.84, 0.93] | 0.88 | [0.83, 0.93] | 0.89 | [0.84, 0.94] | 0.93 | [0.90, 0.97] |
| **SOCIOECONOMIC CHARACTERISTICS** |  |  |  |  |  |  |  |  |  |  |  |  |  |  |  |  |  |  |
| Education level |  |  |  |  |  |  |  |  |  |  |  |  |  |  |  |  |  |  |
| Not literate | 0.65 | [0.50, 0.80] | 0.75 | [0.59, 0.91] | 0.79 | [0.58, 1.00] | 0.65 | [0.50, 0.80] | 0.75 | [0.59, 0.91] | 0.79 | [0.58, 1.00] | 0.78 | [., .] | 0.61 | [., .] | 0.91 | [., .] |
| Primary school | 0.80 | [0.77, 0.84] | 0.86 | [0.83, 0.89] | 0.89 | [0.86, 0.92] | 0.80 | [0.77, 0.84] | 0.86 | [0.83, 0.89] | 0.89 | [0.86, 0.92] | 0.85 | [0.67, 1.03] | 0.88 | [0.72, 1.03] | 0.95 | [0.87, 1.02] |
| Secondary school | 0.82 | [0.78, 0.87] | 0.84 | [0.81, 0.88] | 0.90 | [0.87, 0.93] | 0.82 | [0.78, 0.87] | 0.84 | [0.81, 0.88] | 0.90 | [0.87, 0.93] | 0.94 | [0.90, 0.98] | 0.94 | [0.90, 0.98] | 0.92 | [0.81, 1.03] |
| High school | 0.85 | [0.82, 0.89] | 0.88 | [0.84, 0.91] | 0.92 | [0.90, 0.95] | 0.85 | [0.82, 0.89] | 0.88 | [0.84, 0.91] | 0.92 | [0.90, 0.95] | 0.78 | [0.59, 0.97] | 0.79 | [0.60, 0.99] | 0.88 | [0.74, 1.02] |
| University/Post-graduate | 0.87 | [0.83, 0.91] | 0.90 | [0.87, 0.93] | 0.96 | [0.92, 0.99] | 0.87 | [0.83, 0.91] | 0.90 | [0.87, 0.93] | 0.96 | [0.92, 0.99] | 0.90 | [0.87, 0.94] | 0.95 | [0.92, 0.98] | 0.97 | [0.93, 1.00] |
| Education length |  |  |  |  |  |  |  |  |  |  |  |  |  |  |  |  |  |  |
| 0-4 years | 0.77 | [0.71, 0.83] | 0.85 | [0.79, 0.90] | 0.88 | [0.83, 0.94] | 0.83 | [0.76, 0.90] | 0.79 | [0.67, 0.90] | 0.76 | [0.61, 0.91] | 0.85 | [0.04, 1.65] | 0.79 | [-1.39, 2.96] | 0.96 | [0.39, 1.52] |
| 5-6 years | 0.80 | [0.75, 0.85] | 0.84 | [0.79, 0.89] | 0.87 | [0.81, 0.92] | 0.82 | [0.71, 0.94] | 0.87 | [0.78, 0.96] | 0.87 | [0.78, 0.96] | 0.85 | [0.36, 1.33] | 0.86 | [0.52, 1.20] | 0.92 | [0.76, 1.09] |
| 7-8 years | 0.82 | [0.77, 0.87] | 0.83 | [0.79, 0.88] | 0.88 | [0.83, 0.93] | 0.87 | [0.75, 0.99] | 0.88 | [0.80, 0.97] | 0.86 | [0.75, 0.96] | 0.92 | [0.85, 0.99] | 0.93 | [0.84, 1.01] | 0.95 | [0.89, 1.02] |
| 9-11 years | 0.83 | [0.79, 0.88] | 0.87 | [0.83, 0.90] | 0.91 | [0.88, 0.94] | 0.95 | [0.91, 0.98] | 0.94 | [0.91, 0.97] | 0.98 | [0.96, 1.00] | 0.90 | [0.83, 0.97] | 0.93 | [0.76, 1.09] | 0.86 | [0.24, 1.47] |
| 12+ years | 0.86 | [0.83, 0.89] | 0.89 | [0.87, 0.92] | 0.95 | [0.93, 0.97] | 0.87 | [0.80, 0.93] | 0.89 | [0.83, 0.96] | 0.94 | [0.89, 0.98] | 0.87 | [0.81, 0.93] | 0.90 | [0.84, 0.96] | 0.94 | [0.90, 0.99] |
| Head of household |  |  |  |  |  |  |  |  |  |  |  |  |  |  |  |  |  |  |
| No | 0.82 | [0.79, 0.85] | 0.87 | [0.85, 0.90] | 0.91 | [0.89, 0.94] | 0.82 | [0.72, 0.91] | 0.83 | [0.73, 0.93] | 0.86 | [0.76, 0.96] | 0.89 | [0.84, 0.95] | 0.94 | [0.90, 0.98] | 0.97 | [0.94, 1.00] |
| Yes | 0.82 | [0.80, 0.85] | 0.85 | [0.83, 0.88] | 0.90 | [0.87, 0.92] | 0.89 | [0.85, 0.93] | 0.90 | [0.86, 0.93] | 0.89 | [0.85, 0.94] | 0.86 | [0.79, 0.94] | 0.86 | [0.79, 0.94] | 0.91 | [0.85, 0.97] |
| Household size |  |  |  |  |  |  |  |  |  |  |  |  |  |  |  |  |  |  |
| 0-4 years | 0.85 | [0.80, 0.91] | 0.84 | [0.79, 0.90] | 0.89 | [0.83, 0.96] | 0.95 | [0.85, 1.04] | 0.93 | [0.81, 1.05] | 0.72 | [0.32, 1.12] | 0.61 | [-4.38, 5.60] | 0.62 | [-4.16, 5.40] | 0.87 | [-0.80, 2.54] |
| 5-6 years | 0.75 | [0.68, 0.82] | 0.80 | [0.74, 0.87] | 0.83 | [0.75, 0.90] | 0.89 | [0.82, 0.97] | 0.87 | [0.80, 0.94] | 0.92 | [0.86, 0.97] | 0.92 | [0.83, 1.01] | 0.96 | [0.91, 1.01] | 0.95 | [0.87, 1.04] |
| 7-8 years | 0.83 | [0.78, 0.87] | 0.85 | [0.80, 0.89] | 0.92 | [0.89, 0.95] | 0.81 | [0.68, 0.94] | 0.92 | [0.88, 0.95] | 0.95 | [0.90, 0.99] | 0.89 | [0.83, 0.94] | 0.95 | [0.90, 0.99] | 0.99 | [0.97, 1.01] |
| 9-11 years | 0.83 | [0.79, 0.86] | 0.88 | [0.86, 0.91] | 0.92 | [0.89, 0.95] | 0.95 | [0.91, 0.99] | 0.88 | [0.80, 0.96] | 0.92 | [0.85, 0.99] | 0.87 | [0.76, 0.97] | 0.90 | [0.81, 0.99] | 0.91 | [0.83, 1.00] |
| 12+ years | 0.84 | [0.80, 0.87] | 0.89 | [0.86, 0.91] | 0.92 | [0.90, 0.95] | 0.84 | [0.78, 0.91] | 0.86 | [0.79, 0.93] | 0.85 | [0.77, 0.93] | 0.89 | [0.84, 0.95] | 0.88 | [0.81, 0.95] | 0.93 | [0.86, 1.00] |
| Primary earner |  |  |  |  |  |  |  |  |  |  |  |  |  |  |  |  |  |  |
| No | 0.82 | [0.79, 0.85] | 0.86 | [0.84, 0.89] | 0.90 | [0.87, 0.92] | 0.87 | [0.82, 0.92] | 0.88 | [0.83, 0.92] | 0.87 | [0.82, 0.92] | 0.89 | [0.85, 0.93] | 0.92 | [0.88, 0.95] | 0.94 | [0.89, 0.98] |
| Yes | 0.82 | [0.80, 0.85] | 0.86 | [0.83, 0.88] | 0.91 | [0.89, 0.94] | 0.88 | [0.83, 0.93] | 0.88 | [0.84, 0.92] | 0.92 | [0.85, 0.98] | 0.85 | [0.75, 0.95] | 0.88 | [0.77, 0.98] | 0.94 | [0.90, 0.99] |
| Employment |  |  |  |  |  |  |  |  |  |  |  |  |  |  |  |  |  |  |
| Unemployed | 0.80 | [0.75, 0.85] | 0.85 | [0.80, 0.89] | 0.87 | [0.82, 0.91] | 0.85 | [0.78, 0.92] | 0.84 | [0.77, 0.91] | 0.84 | [0.76, 0.91] | 0.87 | [0.80, 0.95] | 0.89 | [0.83, 0.96] | 0.92 | [0.85, 1.00] |
| Formally employed | 0.86 | [0.82, 0.90] | 0.90 | [0.87, 0.93] | 0.95 | [0.91, 0.98] | 0.85 | [0.63, 1.08] | 0.93 | [0.76, 1.10] | 0.95 | [0.90, 1.01] | 0.92 | [0.85, 1.00] | 0.97 | [0.92, 1.02] | 0.96 | [0.89, 1.02] |
| Informally employed | 0.82 | [0.79, 0.85] | 0.86 | [0.84, 0.89] | 0.91 | [0.89, 0.94] | 0.90 | [0.86, 0.94] | 0.91 | [0.87, 0.95] | 0.93 | [0.87, 1.00] | 0.90 | [0.85, 0.96] | 0.91 | [0.84, 0.98] | 0.93 | [0.88, 0.99] |
| Don't know/No answer | 0.81 | [0.75, 0.86] | 0.83 | [0.78, 0.88] | 0.90 | [0.86, 0.94] | 0.89 | [0.76, 1.01] | 0.90 | [0.85, 0.96] | 0.88 | [0.81, 0.95] | 0.77 | [0.52, 1.02] | 0.81 | [0.55, 1.07] | 0.96 | [0.87, 1.05] |
| Pre-TB monthly income |  |  |  |  |  |  |  |  |  |  |  |  |  |  |  |  |  |  |
| USD 0-24 | 0.80 | [0.75, 0.86] | 0.85 | [0.80, 0.91] | 0.89 | [0.84, 0.94] | 0.82 | [0.74, 0.91] | 0.80 | [0.71, 0.90] | 0.81 | [0.71, 0.91] | 0.90 | [0.83, 0.98] | 0.91 | [0.80, 1.01] | 0.98 | [0.94, 1.01] |
| USD 25-169 | 0.80 | [0.76, 0.85] | 0.83 | [0.79, 0.88] | 0.87 | [0.81, 0.92] | 0.88 | [0.80, 0.95] | 0.90 | [0.86, 0.95] | 0.87 | [0.79, 0.95] | 0.83 | [0.67, 0.99] | 0.86 | [0.73, 1.00] | 0.89 | [0.74, 1.05] |
| USD 170-259 | 0.81 | [0.76, 0.85] | 0.84 | [0.81, 0.88] | 0.89 | [0.85, 0.93] | 0.92 | [0.88, 0.97] | 0.91 | [0.86, 0.95] | 0.97 | [0.94, 0.99] | 0.80 | [0.49, 1.11] | 0.84 | [0.53, 1.15] | 0.93 | [0.82, 1.04] |
| USD 260-389 | 0.84 | [0.80, 0.87] | 0.90 | [0.88, 0.93] | 0.95 | [0.92, 0.97] | 0.92 | [0.85, 0.99] | 0.98 | [0.94, 1.02] | 0.98 | [0.96, 1.00] | 0.88 | [0.81, 0.95] | 0.90 | [0.82, 0.98] | 0.90 | [0.82, 0.99] |
| USD 390+ | 0.84 | [0.80, 0.89] | 0.87 | [0.83, 0.90] | 0.92 | [0.89, 0.96] | 0.88 | [0.79, 0.97] | 0.89 | [0.80, 0.98] | 0.92 | [0.82, 1.02] | 0.92 | [0.87, 0.98] | 0.95 | [0.89, 1.00] | 0.97 | [0.93, 1.01] |
| **SOCIOECONOMIC IMPACT OF TB** |  |  |  |  |  |  |  |  |  |  |  |  |  |  |  |  |  |  |
| Monthly income decline |  |  |  |  |  |  |  |  |  |  |  |  |  |  |  |  |  |  |
| No decline | 0.86 | [0.83, 0.89] | 0.89 | [0.86, 0.92] | 0.93 | [0.90, 0.95] | 0.88 | [0.83, 0.92] | 0.86 | [0.80, 0.91] | 0.87 | [0.81, 0.93] | 0.93 | [0.89, 0.97] | 0.95 | [0.92, 0.98] | 0.96 | [0.91, 1.02] |
| USD 1-100 | 0.78 | [0.72, 0.84] | 0.83 | [0.77, 0.89] | 0.85 | [0.78, 0.92] | 0.83 | [0.71, 0.95] | 0.88 | [0.82, 0.93] | 0.90 | [0.79, 1.00] | 0.83 | [0.67, 1.00] | 0.85 | [0.70, 1.00] | 0.93 | [0.84, 1.02] |
| USD 101-250 | 0.82 | [0.78, 0.87] | 0.84 | [0.80, 0.88] | 0.89 | [0.85, 0.93] | 0.91 | [0.82, 1.00] | 0.94 | [0.87, 1.02] | 0.88 | [0.74, 1.02] | 0.79 | [0.60, 0.97] | 0.81 | [0.62, 1.00] | 0.87 | [0.73, 1.01] |
| USD 251-400 | 0.77 | [0.72, 0.82] | 0.87 | [0.84, 0.90] | 0.92 | [0.88, 0.95] | 0.92 | [0.85, 0.98] | 0.91 | [0.80, 1.02] | 0.96 | [0.92, 1.00] | 0.88 | [0.76, 0.99] | 0.87 | [0.74, 1.00] | 0.94 | [0.86, 1.01] |
| USD 401+ | 0.81 | [0.74, 0.88] | 0.83 | [0.77, 0.88] | 0.89 | [0.84, 0.95] | 0.87 | [0.65, 1.09] | 0.94 | [0.89, 1.00] | 0.89 | [0.64, 1.14] | 0.91 | [0.84, 0.97] | 0.97 | [0.94, 1.00] | 0.98 | [0.94, 1.02] |
| Job loss |  |  |  |  |  |  |  |  |  |  |  |  |  |  |  |  |  |  |
| No | 0.84 | [0.82, 0.86] | 0.87 | [0.85, 0.89] | 0.91 | [0.89, 0.93] | 0.87 | [0.83, 0.91] | 0.87 | [0.84, 0.91] | 0.88 | [0.83, 0.92] | 0.89 | [0.84, 0.93] | 0.91 | [0.87, 0.96] | 0.94 | [0.90, 0.98] |
| Yes | 0.75 | [0.70, 0.80] | 0.83 | [0.79, 0.87] | 0.90 | [0.86, 0.93] | 0.96 | [0.88, 1.04] | 0.97 | [0.88, 1.06] | 0.99 | [0.95, 1.02] | 0.79 | [0.57, 1.00] | 0.76 | [0.41, 1.10] | 0.91 | [0.86, 0.96] |
| Borrow or receive cash |  |  |  |  |  |  |  |  |  |  |  |  |  |  |  |  |  |  |
| No | 0.84 | [0.82, 0.86] | 0.87 | [0.85, 0.89] | 0.91 | [0.89, 0.93] | 0.88 | [0.84, 0.92] | 0.89 | [0.86, 0.92] | 0.89 | [0.85, 0.93] | 0.87 | [0.82, 0.92] | 0.89 | [0.84, 0.94] | 0.93 | [0.89, 0.97] |
| Yes | 0.78 | [0.74, 0.82] | 0.84 | [0.81, 0.87] | 0.89 | [0.85, 0.92] | 0.85 | [0.77, 0.93] | 0.78 | [0.61, 0.95] | 0.84 | [0.70, 0.98] | 0.92 | [0.85, 0.99] | 0.95 | [0.89, 1.00] | 0.96 | [0.91, 1.01] |
| Sell assets |  |  |  |  |  |  |  |  |  |  |  |  |  |  |  |  |  |  |
| No | 0.82 | [0.80, 0.84] | 0.87 | [0.85, 0.88] | 0.90 | [0.89, 0.92] | 0.87 | [0.84, 0.91] | 0.88 | [0.85, 0.92] | 0.89 | [0.85, 0.93] | 0.90 | [0.86, 0.93] | 0.91 | [0.87, 0.95] | 0.94 | [0.91, 0.98] |
| Yes | 0.77 | [0.68, 0.87] | 0.80 | [0.71, 0.88] | 0.91 | [0.86, 0.95] | 0.69 | [., .] | 0.38 | [., .] | 0.03 | [., .] | 0.60 | [0.09, 1.11] | 0.70 | [0.04, 1.35] | 0.90 | [0.62, 1.18] |

Notes: TB=Tuberculosis. ⱡ Includes treatment failure, loss to follow-up, transfer out and death.

**Table 4b. EQ-VAS score by participant characteristics and care pathway and stage of treatment.**

|  | **NTP (N=429)** | | | | | | **ACF (N=106)** | | | | | | **PPM (N=50)** | | | | | |
| --- | --- | --- | --- | --- | --- | --- | --- | --- | --- | --- | --- | --- | --- | --- | --- | --- | --- | --- |
|  | Intensive Phase | | Continuation Phase | | End of Treatment | | Intensive Phase | | Continuation Phase | | End of Treatment | | Intensive Phase | | Continuation Phase | | End of Treatment | |
|  | Mean | 95%CI | Mean | 95%CI | Mean | 95%CI | Mean | 95%CI | Mean | 95%CI | Mean | 95%CI | Mean | 95%CI | Mean | 95%CI | Mean | 95%CI |
| **Total** | 65.5 | [63.7, 67.3] | 70.5 | [68.9, 72.1] | 79.5 | [78.0, 80.9] | 70.4 | [67.1, 73.7] | 69.6 | [66.5, 72.6] | 77.7 | [74.8, 80.7] | 73.9 | [69.2, 78.7] | 76.3 | [71.8, 80.7] | 82.0 | [78.1, 85.9] |
| **DEMOGRAPHICS** |  |  |  |  |  |  |  |  |  |  |  |  |  |  |  |  |  |  |
| Sex |  |  |  |  |  |  |  |  |  |  |  |  |  |  |  |  |  |  |
| Female | 66.8 | [64.8, 68.8] | 71.2 | [69.4, 73.0] | 79.9 | [78.3, 81.6] | 70.9 | [67.1, 74.6] | 70.4 | [67.0, 73.9] | 78.5 | [75.3, 81.7] | 74.3 | [68.1, 80.5] | 74.5 | [68.6, 80.5] | 79.5 | [74.1, 84.9] |
| Male | 61.7 | [58.1, 65.4] | 68.5 | [65.1, 71.8] | 78.0 | [74.9, 81.1] | 67.0 | [59.9, 74.1] | 65.5 | [58.7, 72.3] | 74.5 | [66.4, 82.5] | 73.2 | [65.3, 81.2] | 79.6 | [73.2, 86.0] | 86.8 | [82.0, 91.5] |
| Age |  |  |  |  |  |  |  |  |  |  |  |  |  |  |  |  |  |  |
| <35 years | 73.1 | [70.1, 76.2] | 79.8 | [77.5, 82.2] | 88.3 | [86.4, 90.1] | 81.7 | [74.5, 88.8] | 83.3 | [64.4, 102.3] | 85.0 | [72.6, 97.4] | 75.0 | [67.2, 82.8] | 80.6 | [73.6, 87.6] | 85.3 | [76.8, 93.8] |
| 35-44 years | 65.1 | [61.2, 69.0] | 72.0 | [68.5, 75.5] | 79.8 | [77.0, 82.7] | 67.0 | [46.6, 87.4] | 70.0 | [52.4, 87.6] | 77.0 | [71.4, 82.6] | 71.5 | [59.7, 83.3] | 73.3 | [58.9, 87.7] | 78.0 | [66.4, 89.6] |
| 45-54 years | 64.6 | [60.6, 68.7] | 67.6 | [64.3, 70.8] | 78.6 | [75.2, 81.9] | 73.9 | [66.2, 81.6] | 71.5 | [63.1, 79.9] | 81.5 | [74.7, 88.3] | 84.4 | [73.1, 95.6] | 81.3 | [68.1, 94.4] | 85.6 | [77.4, 93.8] |
| 55-64 years | 61.1 | [57.2, 65.0] | 64.3 | [60.8, 67.7] | 73.3 | [70.1, 76.5] | 72.3 | [66.3, 78.2] | 70.8 | [65.5, 76.1] | 79.5 | [74.2, 84.7] | 75.2 | [66.0, 84.4] | 77.0 | [69.1, 84.9] | 79.5 | [70.2, 88.8] |
| 65+ years | 59.9 | [54.5, 65.3] | 64.9 | [59.7, 70.1] | 73.4 | [68.3, 78.5] | 65.9 | [60.6, 71.1] | 65.9 | [61.3, 70.6] | 73.3 | [68.1, 78.6] | 59.2 | [34.0, 84.3] | 61.7 | [47.7, 75.6] | 79.2 | [69.5, 88.8] |
| City |  |  |  |  |  |  |  |  |  |  |  |  |  |  |  |  |  |  |
| Hanoi | 70.0 | [63.9, 76.1] | 68.1 | [61.7, 74.6] | 75.5 | [69.8, 81.1] | 75.4 | [67.5, 83.4] | 76.3 | [71.0, 81.5] | 77.1 | [68.0, 86.2] | 77.5 | [-17.8, 172.8] | 85.0 | [85.0, 85.0] | 87.5 | [-7.8, 182.8] |
| Hai Phong | 69.8 | [62.6, 77.0] | 70.1 | [64.3, 75.9] | 77.3 | [71.2, 83.5] | 62.5 | [56.0, 68.9] | 59.6 | [53.0, 66.2] | 72.9 | [65.4, 80.3] | 80.1 | [74.2, 86.1] | 78.7 | [71.1, 86.3] | 80.6 | [74.0, 87.2] |
| Ho Chi Minh City | 64.3 | [62.3, 66.2] | 70.6 | [68.9, 72.3] | 79.9 | [78.4, 81.5] | 71.4 | [66.8, 76.1] | 70.3 | [66.2, 74.4] | 78.8 | [74.7, 82.9] | 70.3 | [63.5, 77.1] | 74.4 | [68.4, 80.4] | 82.4 | [77.0, 87.8] |
| Da Nang | 75.2 | [63.7, 86.7] | 77.8 | [71.5, 84.1] | 87.4 | [78.8, 96.0] | 75.8 | [63.7, 87.9] | 80.0 | [72.3, 87.7] | 83.3 | [78.5, 88.1] | . | [., .] | . | [., .] | . | [., .] |
| **CLINICAL CHARACTERISTICS** |  |  |  |  |  |  |  |  |  |  |  |  |  |  |  |  |  |  |
| Diagnosis (N=583) |  |  |  |  |  |  |  |  |  |  |  |  |  |  |  |  |  |  |
| Bacteriologically confirmed | 72.3 | [66.0, 78.6] | 70.8 | [63.4, 78.1] | 78.2 | [71.5, 84.8] | 52.5 | [-42.8, 147.8] | 70.0 | [6.5, 133.5] | 66.5 | [-333.7, 466.7] | 82.0 | [70.9, 93.1] | 83.0 | [76.4, 89.6] | 88.0 | [82.9, 93.1] |
| Clinically diagnosed | 65.0 | [63.1, 66.9] | 70.4 | [68.8, 72.0] | 79.5 | [78.0, 81.0] | 70.7 | [67.4, 74.0] | 69.6 | [66.4, 72.7] | 78.0 | [75.0, 80.9] | 71.9 | [66.6, 77.2] | 74.6 | [69.3, 79.8] | 80.5 | [75.8, 85.2] |
| TB location (N=583) |  |  |  |  |  |  |  |  |  |  |  |  |  |  |  |  |  |  |
| Pulmonary TB | 65.5 | [63.6, 67.3] | 70.4 | [68.8, 72.1] | 79.5 | [78.0, 81.0] | 70.4 | [67.1, 73.7] | 69.6 | [66.5, 72.6] | 77.7 | [74.8, 80.7] | 73.9 | [69.2, 78.7] | 76.3 | [71.8, 80.7] | 82.0 | [78.1, 85.9] |
| Extrapulmonary TB | 65.0 | [50.9, 79.1] | 70.0 | [58.2, 81.8] | 75.9 | [64.8, 87.0] | . | [., .] | . | [., .] | . | [., .] | . | [., .] | . | [., .] | . | [., .] |
| Treatment category (N=583) |  |  |  |  |  |  |  |  |  |  |  |  |  |  |  |  |  |  |
| New | 65.7 | [63.7, 67.8] | 70.9 | [69.0, 72.7] | 79.5 | [77.8, 81.2] | 70.4 | [67.1, 73.7] | 69.6 | [66.5, 72.6] | 77.7 | [74.8, 80.7] | 74.5 | [69.7, 79.4] | 76.6 | [72.1, 81.2] | 82.6 | [78.6, 86.6] |
| Retreatment | 64.6 | [61.0, 68.1] | 69.0 | [65.9, 72.1] | 79.1 | [76.3, 81.9] | . | [., .] | . | [., .] | . | [., .] | 60.0 | [60.0, 60.0] | 67.5 | [-27.8, 162.8] | 67.5 | [-27.8, 162.8] |
| Treatment outcome (N=531) |  |  |  |  |  |  |  |  |  |  |  |  |  |  |  |  |  |  |
| Treatment success | 65.3 | [63.4, 67.2] | 70.6 | [68.9, 72.3] | 79.7 | [78.2, 81.2] | 72.7 | [68.7, 76.6] | 72.2 | [68.8, 75.6] | 79.4 | [75.9, 82.8] | 73.9 | [69.2, 78.7] | 76.3 | [71.8, 80.7] | 82.0 | [78.1, 85.9] |
| Unfavorable outcomeⱡ | 63.2 | [56.1, 70.2] | 67.4 | [58.7, 76.1] | 74.2 | [66.7, 81.7] | 74.0 | [60.5, 87.5] | 76.0 | [59.3, 92.7] | 79.0 | [69.8, 88.2] | . | [., .] | . | [., .] | . | [., .] |
| **HEALTH ACCESS** |  |  |  |  |  |  |  |  |  |  |  |  |  |  |  |  |  |  |
| Diagnostic delay (N=515) |  |  |  |  |  |  |  |  |  |  |  |  |  |  |  |  |  |  |
| 1-3 weeks | 70.2 | [66.0, 74.4] | 73.3 | [69.5, 77.2] | 83.9 | [80.9, 86.9] | 67.5 | [51.7, 83.3] | 74.2 | [57.8, 90.6] | 65.0 | [44.5, 85.5] | 78.8 | [67.0, 90.5] | 82.5 | [71.6, 93.4] | 83.8 | [71.0, 96.5] |
| 4-5 weeks | 70.0 | [65.5, 74.5] | 73.8 | [69.9, 77.6] | 80.2 | [76.6, 83.7] | 76.3 | [65.7, 86.8] | 71.3 | [57.8, 84.7] | 75.0 | [61.1, 88.9] | 75.0 | [61.1, 88.9] | 71.4 | [56.6, 86.2] | 80.0 | [66.7, 93.3] |
| 6-10 weeks | 65.8 | [61.8, 69.7] | 71.1 | [67.4, 74.8] | 80.8 | [78.0, 83.5] | 76.3 | [65.5, 87.0] | 68.3 | [58.8, 77.8] | 79.8 | [72.7, 86.9] | 67.3 | [53.1, 81.5] | 72.3 | [60.1, 84.6] | 84.6 | [76.4, 92.8] |
| 11-21 weeks | 59.6 | [55.8, 63.4] | 67.3 | [63.8, 70.7] | 76.5 | [72.9, 80.1] | 69.5 | [57.3, 81.7] | 66.1 | [59.9, 72.3] | 77.9 | [70.4, 85.5] | 80.0 | [71.9, 88.1] | 81.4 | [72.1, 90.6] | 80.5 | [70.9, 90.0] |
| 22+ weeks | 62.5 | [58.5, 66.5] | 67.9 | [64.4, 71.3] | 75.6 | [71.9, 79.3] | 68.3 | [60.1, 76.4] | 64.7 | [57.2, 72.2] | 75.6 | [67.4, 83.9] | 70.8 | [60.3, 81.3] | 75.3 | [69.6, 81.0] | 79.4 | [68.0, 90.9] |
| Health-seeking |  |  |  |  |  |  |  |  |  |  |  |  |  |  |  |  |  |  |
| 1-2 attempts | 67.0 | [59.8, 74.2] | 72.9 | [67.8, 78.0] | 81.4 | [75.3, 87.4] | 69.2 | [63.9, 74.6] | 68.4 | [63.2, 73.6] | 79.2 | [74.4, 84.0] | 75.0 | [59.1, 90.9] | 69.1 | [55.0, 83.2] | 73.6 | [63.1, 84.2] |
| 3-4 attempts | 65.9 | [61.6, 70.2] | 70.7 | [67.2, 74.2] | 79.7 | [76.9, 82.5] | 73.3 | [67.7, 78.8] | 73.4 | [68.4, 78.3] | 79.0 | [73.8, 84.2] | 76.2 | [68.3, 84.2] | 83.9 | [77.1, 90.7] | 85.7 | [80.7, 90.8] |
| 5-6 attempts | 67.6 | [63.4, 71.8] | 72.6 | [69.2, 76.0] | 80.4 | [77.2, 83.6] | 64.6 | [53.2, 76.1] | 66.1 | [55.1, 77.1] | 67.5 | [59.7, 75.3] | 77.7 | [66.7, 88.8] | 76.4 | [65.1, 87.6] | 82.7 | [72.4, 93.1] |
| 7-10 attempts | 65.6 | [62.1, 69.2] | 69.0 | [65.2, 72.8] | 78.3 | [75.2, 81.5] | 67.4 | [51.3, 83.5] | 62.0 | [47.7, 76.3] | 79.0 | [67.9, 90.1] | 74.5 | [70.9, 78.1] | 76.5 | [71.4, 81.6] | 88.0 | [82.9, 93.1] |
| 11+ attempts | 63.0 | [59.9, 66.1] | 69.3 | [66.5, 72.0] | 79.0 | [76.2, 81.9] | 71.0 | [58.4, 83.6] | 64.4 | [55.9, 72.9] | 81.6 | [69.7, 93.6] | 51.3 | [34.8, 67.7] | 68.3 | [43.0, 93.5] | 75.0 | [38.8, 111.2] |
| Social Health Insurance |  |  |  |  |  |  |  |  |  |  |  |  |  |  |  |  |  |  |
| No | 62.5 | [58.8, 66.2] | 70.6 | [67.4, 73.9] | 80.1 | [77.4, 82.9] | 71.3 | [62.1, 80.5] | 70.8 | [63.4, 78.2] | 74.2 | [67.4, 80.9] | 77.5 | [63.9, 91.1] | 83.8 | [73.8, 93.7] | 85.6 | [70.2, 101.0] |
| Yes | 66.5 | [64.5, 68.6] | 70.4 | [68.6, 72.2] | 79.2 | [77.5, 81.0] | 70.2 | [66.6, 73.8] | 69.3 | [65.9, 72.7] | 78.5 | [75.1, 81.8] | 73.3 | [68.0, 78.5] | 74.8 | [69.9, 79.8] | 81.3 | [77.3, 85.3] |
| **SOCIOECONOMIC CHARACTERISTICS** |  |  |  |  |  |  |  |  |  |  |  |  |  |  |  |  |  |  |
| Education level |  |  |  |  |  |  |  |  |  |  |  |  |  |  |  |  |  |  |
| Not literate | 46.9 | [35.1, 58.8] | 67.8 | [59.8, 75.7] | 75.9 | [68.7, 83.1] | 58.3 | [36.9, 79.8] | 58.3 | [34.9, 81.7] | 66.7 | [39.6, 93.8] | 55.0 | [., .] | 80.0 | [., .] | 70.0 | [., .] |
| Primary school | 62.8 | [60.2, 65.4] | 68.3 | [65.7, 70.8] | 77.7 | [75.3, 80.2] | 65.8 | [60.5, 71.2] | 66.2 | [62.0, 70.4] | 75.2 | [70.6, 79.8] | 74.6 | [60.8, 88.5] | 71.3 | [59.7, 82.8] | 81.3 | [68.9, 93.6] |
| Secondary school | 64.6 | [61.0, 68.3] | 68.1 | [65.0, 71.3] | 77.4 | [74.5, 80.3] | 75.7 | [70.0, 81.4] | 72.5 | [66.6, 78.5] | 81.3 | [76.3, 86.3] | 77.0 | [65.9, 88.1] | 74.8 | [64.6, 85.0] | 76.5 | [67.0, 86.0] |
| High school | 71.9 | [67.8, 75.9] | 73.2 | [69.3, 77.0] | 81.4 | [78.0, 84.7] | 74.3 | [68.9, 79.7] | 74.4 | [67.3, 81.6] | 80.7 | [75.2, 86.2] | 67.2 | [48.4, 86.1] | 76.1 | [58.8, 93.4] | 80.6 | [66.9, 94.2] |
| University/Post-graduate | 72.0 | [67.9, 76.1] | 79.0 | [75.6, 82.4] | 87.3 | [84.8, 89.8] | 71.7 | [47.1, 96.2] | 73.3 | [57.2, 89.5] | 77.5 | [54.3, 100.7] | 75.9 | [70.0, 81.9] | 78.6 | [72.3, 85.0] | 85.9 | [80.7, 91.1] |
| Education length |  |  |  |  |  |  |  |  |  |  |  |  |  |  |  |  |  |  |
| 0-4 years | 58.5 | [54.4, 62.7] | 68.8 | [65.3, 72.3] | 78.3 | [75.0, 81.7] | 64.5 | [56.8, 72.3] | 61.9 | [54.6, 69.2] | 73.8 | [65.3, 82.3] | 66.0 | [-73.8, 205.8] | 75.0 | [11.5, 138.5] | 75.0 | [11.5, 138.5] |
| 5-6 years | 63.2 | [59.2, 67.1] | 66.5 | [62.2, 70.7] | 76.7 | [72.8, 80.6] | 63.8 | [54.8, 72.7] | 67.8 | [61.2, 74.3] | 72.0 | [65.2, 78.8] | 75.0 | [35.0, 115.0] | 76.3 | [47.7, 104.8] | 85.0 | [73.7, 96.3] |
| 7-8 years | 60.8 | [55.8, 65.8] | 65.8 | [61.6, 69.9] | 73.7 | [69.5, 77.9] | 68.9 | [62.2, 75.6] | 63.5 | [56.4, 70.6] | 74.8 | [69.1, 80.5] | 81.5 | [74.5, 88.5] | 75.0 | [66.2, 83.8] | 81.0 | [71.2, 90.8] |
| 9-11 years | 67.5 | [63.8, 71.3] | 69.8 | [66.5, 73.1] | 78.4 | [75.3, 81.5] | 78.1 | [72.0, 84.3] | 77.4 | [72.1, 82.6] | 83.8 | [77.3, 90.3] | 58.3 | [39.4, 77.3] | 64.3 | [22.3, 106.4] | 61.7 | [30.4, 92.9] |
| 12+ years | 72.9 | [69.8, 76.0] | 77.1 | [74.4, 79.8] | 85.8 | [83.7, 87.9] | 75.5 | [67.4, 83.7] | 78.0 | [70.9, 85.1] | 84.5 | [80.6, 88.4] | 73.4 | [67.0, 79.8] | 77.9 | [71.8, 84.0] | 84.4 | [79.3, 89.4] |
| Head of household |  |  |  |  |  |  |  |  |  |  |  |  |  |  |  |  |  |  |
| No | 66.8 | [64.3, 69.4] | 72.6 | [70.4, 74.9] | 81.0 | [79.0, 83.0] | 67.6 | [60.2, 75.0] | 68.0 | [62.4, 73.7] | 76.9 | [71.9, 81.9] | 75.2 | [67.9, 82.5] | 80.1 | [74.9, 85.4] | 85.0 | [79.5, 90.5] |
| Yes | 64.4 | [61.9, 66.9] | 68.6 | [66.4, 70.8] | 78.1 | [76.1, 80.2] | 71.3 | [67.6, 75.1] | 70.1 | [66.4, 73.8] | 78.0 | [74.3, 81.7] | 72.7 | [66.2, 79.2] | 72.4 | [65.2, 79.6] | 79.0 | [73.3, 84.7] |
| Household size |  |  |  |  |  |  |  |  |  |  |  |  |  |  |  |  |  |  |
| 0-4 years | 68.3 | [62.1, 74.4] | 67.4 | [60.3, 74.4] | 78.9 | [73.1, 84.6] | 60.8 | [41.4, 80.3] | 75.0 | [59.1, 90.9] | 76.7 | [56.0, 97.3] | 57.5 | [-164.9, 279.9] | 55.0 | [-262.7, 372.7] | 65.0 | [-125.6, 255.6] |
| 5-6 years | 62.5 | [57.5, 67.4] | 65.6 | [61.6, 69.6] | 74.6 | [70.3, 79.0] | 68.4 | [59.2, 77.7] | 69.7 | [62.2, 77.3] | 74.6 | [68.0, 81.2] | 77.9 | [62.3, 93.4] | 80.0 | [65.1, 94.9] | 87.9 | [79.9, 95.8] |
| 7-8 years | 64.0 | [60.2, 67.9] | 68.6 | [65.2, 72.0] | 80.6 | [77.5, 83.6] | 69.3 | [60.2, 78.5] | 67.2 | [58.6, 75.7] | 81.3 | [73.7, 88.9] | 79.1 | [70.2, 88.0] | 83.6 | [79.1, 88.2] | 90.5 | [86.4, 94.6] |
| 9-11 years | 67.2 | [63.7, 70.7] | 71.8 | [68.8, 74.8] | 80.2 | [77.4, 83.1] | 72.1 | [64.8, 79.4] | 74.7 | [69.2, 80.3] | 81.8 | [73.2, 90.5] | 72.3 | [62.4, 82.2] | 72.7 | [61.8, 83.6] | 79.6 | [69.0, 90.3] |
| 12+ years | 65.9 | [62.6, 69.2] | 73.9 | [71.1, 76.7] | 80.7 | [78.3, 83.1] | 72.1 | [67.0, 77.2] | 67.6 | [62.4, 72.7] | 76.0 | [71.5, 80.5] | 72.2 | [62.8, 81.6] | 75.2 | [68.2, 82.1] | 77.9 | [71.6, 84.2] |
| Primary earner |  |  |  |  |  |  |  |  |  |  |  |  |  |  |  |  |  |  |
| No | 64.8 | [62.3, 67.3] | 70.7 | [68.5, 72.9] | 79.5 | [77.5, 81.5] | 69.9 | [65.9, 73.9] | 69.1 | [65.3, 72.8] | 76.4 | [72.6, 80.1] | 75.5 | [69.6, 81.5] | 76.8 | [71.8, 81.9] | 81.7 | [76.9, 86.6] |
| Yes | 66.3 | [63.7, 68.8] | 70.3 | [68.0, 72.5] | 79.4 | [77.3, 81.6] | 71.5 | [65.2, 77.8] | 70.8 | [65.3, 76.4] | 81.3 | [76.3, 86.2] | 71.1 | [62.6, 79.6] | 75.3 | [66.2, 84.4] | 82.5 | [75.1, 89.9] |
| Employment |  |  |  |  |  |  |  |  |  |  |  |  |  |  |  |  |  |  |
| Unemployed | 64.5 | [60.6, 68.5] | 68.3 | [64.8, 71.7] | 77.7 | [74.7, 80.8] | 66.7 | [61.3, 72.0] | 65.3 | [61.1, 69.6] | 74.1 | [69.2, 79.1] | 73.4 | [65.6, 81.1] | 74.1 | [66.3, 81.9] | 81.8 | [75.3, 88.3] |
| Formally employed | 72.0 | [68.0, 76.0] | 76.1 | [72.5, 79.7] | 85.0 | [81.2, 88.7] | 78.0 | [70.9, 85.1] | 76.0 | [61.8, 90.2] | 82.0 | [64.1, 99.9] | 78.3 | [68.0, 88.7] | 82.2 | [73.6, 90.8] | 83.3 | [71.3, 95.3] |
| Informally employed | 64.4 | [61.8, 67.0] | 71.5 | [69.3, 73.7] | 79.9 | [77.9, 81.9] | 74.9 | [69.7, 80.0] | 74.4 | [69.6, 79.3] | 81.9 | [77.6, 86.2] | 73.2 | [64.0, 82.5] | 77.6 | [69.9, 85.4] | 83.5 | [77.6, 89.5] |
| Don't know/No answer | 64.3 | [59.5, 69.0] | 63.9 | [59.3, 68.5] | 74.3 | [70.4, 78.2] | 68.1 | [58.9, 77.3] | 68.4 | [57.5, 79.3] | 77.1 | [68.4, 85.7] | 71.4 | [50.3, 92.6] | 70.4 | [50.4, 90.5] | 77.1 | [58.3, 96.0] |
| Pre-TB monthly income |  |  |  |  |  |  |  |  |  |  |  |  |  |  |  |  |  |  |
| USD 0-24 | 62.6 | [58.0, 67.1] | 67.2 | [63.1, 71.3] | 76.5 | [72.8, 80.1] | 63.9 | [57.8, 70.1] | 60.9 | [55.6, 66.3] | 72.4 | [66.0, 78.8] | 74.4 | [57.9, 90.9] | 81.3 | [69.7, 92.8] | 81.3 | [68.9, 93.6] |
| USD 25-169 | 64.6 | [60.2, 69.1] | 68.4 | [64.4, 72.4] | 77.7 | [74.2, 81.2] | 72.2 | [66.5, 78.0] | 74.0 | [68.5, 79.5] | 80.1 | [74.9, 85.3] | 74.6 | [61.1, 88.1] | 71.3 | [58.1, 84.4] | 80.6 | [72.1, 89.1] |
| USD 170-259 | 63.8 | [60.2, 67.5] | 69.4 | [66.1, 72.7] | 79.7 | [76.6, 82.8] | 77.8 | [70.5, 85.1] | 72.5 | [66.8, 78.2] | 82.9 | [76.9, 89.0] | 71.7 | [49.2, 94.1] | 75.8 | [49.4, 102.3] | 79.2 | [61.8, 96.5] |
| USD 260-389 | 67.7 | [63.8, 71.5] | 73.8 | [70.4, 77.1] | 81.5 | [78.3, 84.7] | 71.5 | [61.4, 81.6] | 77.5 | [67.2, 87.8] | 76.0 | [70.2, 81.8] | 69.6 | [58.8, 80.5] | 73.5 | [64.2, 82.7] | 80.4 | [71.2, 89.6] |
| USD 390+ | 68.7 | [64.9, 72.4] | 72.9 | [69.7, 76.1] | 81.1 | [78.2, 84.0] | 70.5 | [55.6, 85.4] | 69.5 | [57.5, 81.5] | 79.5 | [68.0, 91.0] | 78.0 | [70.8, 85.2] | 78.9 | [71.9, 85.8] | 85.7 | [78.1, 93.3] |
| **SOCIOECONOMIC IMPACT OF TB** |  |  |  |  |  |  |  |  |  |  |  |  |  |  |  |  |  |  |
| Monthly income decline |  |  |  |  |  |  |  |  |  |  |  |  |  |  |  |  |  |  |
| No decline | 66.4 | [63.5, 69.3] | 71.9 | [69.2, 74.5] | 81.2 | [79.0, 83.4] | 71.2 | [66.8, 75.6] | 69.9 | [65.2, 74.6] | 77.8 | [73.7, 81.9] | 79.7 | [71.7, 87.7] | 82.4 | [75.5, 89.2] | 85.9 | [79.3, 92.5] |
| USD 1-100 | 63.5 | [58.6, 68.4] | 67.7 | [63.0, 72.4] | 78.6 | [74.4, 82.7] | 70.1 | [62.9, 77.2] | 68.4 | [62.7, 74.1] | 77.6 | [70.0, 85.3] | 72.8 | [53.9, 91.6] | 73.8 | [57.8, 89.7] | 78.1 | [64.9, 91.3] |
| USD 101-250 | 65.9 | [61.7, 70.0] | 68.6 | [64.7, 72.4] | 76.9 | [73.4, 80.4] | 73.9 | [62.4, 85.4] | 71.4 | [60.3, 82.4] | 79.1 | [70.8, 87.4] | 72.2 | [61.0, 83.4] | 68.9 | [52.7, 85.1] | 72.2 | [60.4, 84.1] |
| USD 251-400 | 64.9 | [60.4, 69.3] | 72.4 | [69.1, 75.8] | 79.8 | [75.9, 83.7] | 60.0 | [43.3, 76.7] | 68.1 | [59.2, 77.0] | 77.5 | [68.0, 87.0] | 66.3 | [51.3, 81.2] | 71.9 | [61.7, 82.1] | 80.6 | [72.4, 88.8] |
| USD 401+ | 65.7 | [60.5, 70.9] | 69.1 | [64.8, 73.3] | 78.1 | [74.1, 82.1] | 71.0 | [46.8, 95.2] | 70.0 | [50.4, 89.6] | 75.0 | [50.2, 99.8] | 72.5 | [61.8, 83.2] | 78.5 | [71.0, 86.0] | 90.0 | [82.3, 97.7] |
| Job loss |  |  |  |  |  |  |  |  |  |  |  |  |  |  |  |  |  |  |
| No | 67.1 | [65.1, 69.1] | 70.7 | [68.8, 72.6] | 80.0 | [78.3, 81.6] | 70.5 | [67.1, 73.8] | 69.4 | [66.3, 72.6] | 77.7 | [74.6, 80.8] | 75.7 | [71.2, 80.2] | 76.9 | [72.2, 81.6] | 82.3 | [78.2, 86.4] |
| Yes | 60.5 | [56.7, 64.3] | 69.8 | [67.0, 72.5] | 77.9 | [74.7, 81.1] | 68.0 | [41.1, 94.9] | 72.0 | [55.8, 88.2] | 79.0 | [59.7, 98.3] | 53.8 | [20.4, 87.1] | 68.8 | [47.8, 89.7] | 78.8 | [55.9, 101.6] |
| Borrow or receive cash |  |  |  |  |  |  |  |  |  |  |  |  |  |  |  |  |  |  |
| No | 66.6 | [64.4, 68.7] | 71.2 | [69.3, 73.1] | 80.8 | [79.1, 82.5] | 71.2 | [67.7, 74.8] | 70.4 | [67.1, 73.6] | 77.6 | [74.3, 80.9] | 73.7 | [68.2, 79.2] | 75.7 | [70.4, 81.0] | 80.9 | [76.2, 85.5] |
| Yes | 63.4 | [60.1, 66.6] | 68.9 | [66.1, 71.7] | 76.6 | [73.9, 79.4] | 64.1 | [55.0, 73.2] | 63.8 | [54.9, 72.8] | 78.8 | [72.3, 85.4] | 75.0 | [64.0, 86.0] | 78.9 | [72.3, 85.5] | 87.2 | [81.1, 93.3] |
| Sell assets |  |  |  |  |  |  |  |  |  |  |  |  |  |  |  |  |  |  |
| No | 66.0 | [64.2, 67.9] | 71.0 | [69.4, 72.6] | 79.8 | [78.3, 81.3] | 70.5 | [67.1, 73.8] | 69.8 | [66.7, 72.8] | 77.9 | [74.9, 80.9] | 75.7 | [71.4, 80.0] | 77.1 | [72.7, 81.5] | 81.9 | [77.8, 86.0] |
| Yes | 57.7 | [50.4, 65.0] | 62.0 | [54.0, 70.1] | 74.4 | [69.2, 79.6] | 60.0 | [., .] | 50.0 | [., .] | 60.0 | [., .] | 46.7 | [-17.1, 110.4] | 63.3 | [6.0, 120.7] | 83.3 | [45.4, 121.3] |

Notes: TB=Tuberculosis. ⱡ Includes treatment failure, loss to follow-up, transfer out and death.
